# Three‐year follow‐up results from phase II studies of nivolumab in Japanese patients with previously treated advanced non‐small cell lung cancer: Pooled analysis of ONO‐4538‐05 and ONO‐4538‐06 studies

**DOI:** 10.1002/cam4.2411

**Published:** 2019-07-29

**Authors:** Hidehito Horinouchi, Makoto Nishio, Toyoaki Hida, Kazuhiko Nakagawa, Hiroshi Sakai, Naoyuki Nogami, Shinji Atagi, Toshiaki Takahashi, Hideo Saka, Mitsuhiro Takenoyama, Nobuyuki Katakami, Hiroshi Tanaka, Koji Takeda, Miyako Satouchi, Hiroshi Isobe, Makoto Maemondo, Koichi Goto, Tomonori Hirashima, Koichi Minato, Naoki Sumiyoshi, Tomohide Tamura

**Affiliations:** ^1^ Department of Thoracic Oncology National Cancer Center Hospital Tokyo Japan; ^2^ Department of Thoracic Medical Oncology Cancer Institute Hospital Tokyo Japan; ^3^ Department of Thoracic Oncology Aichi Cancer Center Hospital Nagoya Japan; ^4^ Department of Medical Oncology, Faculty of Medicine Kindai University Osaka Japan; ^5^ Department of Thoracic Oncology Saitama Cancer Center Saitama Japan; ^6^ Division of Thoracic Oncology and Medicine National Hospital Organization Shikoku Cancer Center Ehime Japan; ^7^ Department of Internal Medicine Kinki‐Chuo Chest Medical Center Sakai Japan; ^8^ Division of Thoracic Oncology Shizuoka Cancer Center Shizuoka Japan; ^9^ Department of Medical Oncology Nagoya Medical Center Nagoya Japan; ^10^ Department of Thoracic Oncology National Hospital Organization Kyushu Cancer Center Fukuoka Japan; ^11^ Department of Medical Oncology Kobe City Medical Center Kobe Japan; ^12^ Department of Internal Medicine Niigata Cancer Center Hospital Niigata Japan; ^13^ Department of Medical Oncology Osaka City General Hospital Osaka Japan; ^14^ Department of Thoracic Oncology Hyogo Cancer Center Hyogo Japan; ^15^ Department of Medical Oncology KKR Sapporo Medical Center Sapporo Japan; ^16^ Division of Pulmonary Medicine, Allergy and Rheumatology, Department of Internal Medicine Iwate Medical University School of Medicine Iwate Japan; ^17^ Department of Respiratory Medicine Miyagi Cancer Center Miyagi Japan; ^18^ Division of Thoracic Oncology National Cancer Center Hospital East Chiba Japan; ^19^ Department of Thoracic Oncology Osaka Habikino Medical Center Osaka Japan; ^20^ Division of Respiratory Medicine Gunma Prefectural Cancer Center Gunma Japan; ^21^ Oncology Clinical Development Planning Ono Pharmaceutical Co., Ltd Osaka Japan; ^22^ Thoracic Center St. Luke's International Hospital Tokyo Japan

**Keywords:** nivolumab, non‐small cell lung cancer (NSCLC), phase II study, programmed cell death 1 ligand 1 (PD‐L1), programmed cell death 1 receptor (PD‐1)

## Abstract

**Background:**

Nivolumab is a programmed cell death 1 (PD‐1) receptor inhibitor antibody that enhances immune system antitumor activity. It is associated with longer overall survival (OS) than the standard treatment of docetaxel in patients with previously treated advanced squamous (SQ) and non‐squamous (non‐SQ) non‐small cell lung cancer (NSCLC). We previously conducted two phase II studies of nivolumab in Japanese patients with SQ (ONO‐4538‐05) and non‐SQ (ONO‐4538‐06) NSCLC, showing overall response rates (ORRs) (95% CI) of 25.7% (14.2‐42.1) and 22.4% (14.5‐32.9), respectively, with acceptable toxicity. In this analysis, we more precisely estimated the long‐term safety and efficacy in patients with SQ and non‐SQ NSCLC by pooling data from these two trials.

**Methods:**

SQ (N = 35) and non‐SQ (N = 76) NSCLC patients received nivolumab (3 mg/kg, every 2 weeks) until progression or discontinuation. OS was estimated using the Kaplan–Meier method. A pooled analysis of SQ and non‐SQ patients was also performed.

**Results:**

In SQ NSCLC patients, the median OS (95% CI) was 16.3 months (12.4‐25.2), and the estimated 1‐year, 2‐year, and 3‐year survival rates were 71.4% (53.4‐83.5), 37.1% (21.6‐52.7), and 20.0% (8.8‐34.4), respectively. In non‐SQ NSCLC patients, median OS was 17.1 months (13.3‐23.0), and the estimated 1‐, 2‐, and 3‐year survival rates were 68.0% (56.2‐77.3), 37.4% (26.5‐48.1), and 31.9% (21.7‐42.5), respectively. When SQ NSCLC and non‐SQ NSCLC data were pooled, the median OS was 17.1 months (14.2‐20.6), and the estimated 1‐, 2‐, and 3‐year survival rates were 69.1% (59.6‐76.8), 37.3% (28.3‐46.2), and 28.1% (20.0‐36.7), respectively. Twenty (76.9%) of 26 responders lived for 3 or more years. Nivolumab was well tolerated and no new safety signals were found.

**Conclusion:**

Treatment with nivolumab improved long‐term survival and was well tolerated in patients with SQ and non‐SQ NSCLC.

**Trial registration:**

JapicCTI‐132072; JapicCTI‐132073.

## 1 INTRODUCTION

Lung cancer is among the leading causes of cancer‐related deaths globally.^1^ Non‐small cell lung cancer (NSCLC) accounts for 85%‐90% of all lung cancers and can be histologically subclassified as squamous (SQ) or non‐squamous (non‐SQ).^2^ Limited effective treatment options exist for patients with NSCLC whose disease progresses after first‐line chemotherapy. Docetaxel is currently approved as second‐line treatment for advanced NSCLC based on the longer survival than that observed with best supportive care. Newer chemotherapeutic agents, including pemetrexed and erlotinib, are associated with fewer side effects than docetaxel but have been unable to show superiority or noninferiority to docetaxel with respect to overall survival (OS) when used as second‐line therapy.^3^ However, treatment strategies for patients with advanced NSCLC have been revolutionized by the recent development of novel immunotherapeutic drugs with various mechanisms of action, including angiogenesis, immune checkpoint, and epidermal growth factor receptor (EGFR) inhibitors.^4^

The programmed cell death 1 (PD‐1) receptor, a T‐cell checkpoint receptor protein, suppresses antitumor immunity in a number of malignancies, including NSCLC,^5^ and tumor expression of PD‐L1, an endogenous ligand of PD‐1, is associated with a poor prognosis.^6^ Nivolumab, a fully human PD‐1 immune checkpoint inhibitor antibody that disrupts PD‐1–mediated signaling and may restore antitumor immunity, is approved for the treatment of patients with metastatic NSCLC and disease progression on or after platinum‐based chemotherapy. In a phase I study, nivolumab monotherapy showed durable antitumor activity and encouraging results on survival in all NSCLC subtypes. Among heavily pretreated patients with advanced non‐SQ NSCLC, nivolumab was associated with a response rate of 17.6%; OS rates of 42% at 1 year, 23% at 2 years, and 16% at 3 years; and a progression‐free survival (PFS) rate of 18% at 1 year.^7^ In two phase III studies in patients with NSCLC who progressed after first‐line platinum‐based doublet chemotherapy, nivolumab conferred a survival benefit over docetaxel in patients previously treated for advanced SQ and non‐SQ NSCLC.^3^, ^8^

Although these results indicate that nivolumab is efficacious in patients with SQ and non‐SQ NSCLC, clinical data in Japanese patients are limited, and few reports of long‐term efficacy and safety of immune checkpoint inhibitors in general have been published to date. Accordingly, we performed two multicenter phase II studies of nivolumab in Japanese patients with SQ (ONO‐4538‐05) and non‐SQ (ONO‐4538‐06) NSCLC^9^, ^10^ showing primary endpoints of independent radiology review committee (IRC)‐assessed overall response rates (ORRs) of 25.7% (95%CI 14.2‐42.1) and 22.4% (95%CI 14.5‐32.9), respectively, with acceptable toxicity. The clinical efficacy and manageable tolerability demonstrated in these phase II studies support the use of nivolumab in Japanese patients with advanced or recurrent SQ and non‐SQ NSCLC. The present analysis updates the long‐term safety and efficacy results of the ONO‐4538‐05 and ONO‐4538‐06 studies, with a focus on OS, and includes a subgroup analysis of tumor PD‐L1 expression status in Japanese patients with advanced or recurrent SQ and non‐SQ NSCLC at a median follow‐up of approximately 3 years.

## 2 METHODS

### 2.1 Patients

The full eligibility criteria for both the ONO‐4538‐05 and ONO‐4538‐06 studies have been previously described.^9^, ^10^ Briefly, patients were ≥20 years of age, had an Eastern Cooperative Oncology Group performance status (ECOG PS) of 0 or 1, and had histologically or cytologically confirmed SQ/non‐SQ NSCLC, stage IIIB/IV disease (according to the Union for International Cancer Control TNM 7th edition classification^11^, ^12^), or recurrent SQ/non‐SQ NSCLC after surgical resection.

### 2.2 Study design

ONO‐4538‐05 and ONO‐4538‐06 were multicenter, open‐label, phase II studies conducted in Japanese patients with SQ and non‐SQ NSCLC, respectively. ONO‐4538‐05 enrolled 35 patients from 17 sites in Japan between May 2013 and April 2014, while ONO‐4538‐06 enrolled 76 patients from 19 sites in Japan between April and October 2013. The data cut‐off for the 3‐year follow‐up period for both studies was 31 December 2017. In each trial, patients were administered nivolumab 3 mg/kg intravenously every 2 weeks in each 6‐week cycle until radiologically confirmed progressive disease (PD), unacceptable toxicity, withdrawal, or death. The studies were conducted in accordance with the ethical principles of the Declaration of Helsinki and are registered on the Japan Pharmaceutical Information Center—Clinical Trials Information (JapicCTI) registry (ONO‐4538‐05, JapicCTI‐132072; ONO‐4538‐06, JapicCTI‐132073), and the study protocols were approved by the institutional review board at each site. All patients provided written informed consent prior to participation.

### 2.3 Assessments

The primary efficacy endpoint was IRC‐assessed confirmed ORR, which was evaluated based on tumor response assessed according to RECIST guidelines (version 1.1). ORR was calculated as the proportion of patients with a best overall response (BOR) of complete response or partial response. Secondary efficacy endpoints included OS, PFS, duration of response (DOR), and BOR. Adverse events (AEs) were graded according to the National Cancer Institute Common Terminology Criteria for Adverse Events, version 4.0,^13^ and selected AEs with a potential immunologic cause (immune‐related AEs; irAEs) were grouped according to prespecified categories. As a biomarker analysis, tumor PD‐L1 expression was assessed retrospectively in the primary studies in pretreatment tumor‐biopsy specimens by immunohistochemistry, as described in the primary analyses. The association of PD‐L1 expression with OS was subsequently evaluated in the current analysis.

### 2.4 Statistical analysis

The sample size for both primary studies was calculated based on the threshold response rate for nivolumab, and efficacy and safety were assessed in all patients who received at least one dose of nivolumab, as described in the primary analyses.^9^, ^10^ For this 3‐year update, a safety analysis was conducted using pooled data from both trials. Survival and PFS curves and rates were estimated using the Kaplan–Meier method. The chi‐squared or Wilcoxon signed‐rank tests were used to compare the nonparametric variables. P‐values of less than 0.05 were considered statistically significant.

## 3 RESULTS

### 3.1 Patients and treatment

The baseline characteristics of patients from the ONO‐4538‐05 and ONO‐4538‐06 studies are shown in Table 1. Following enrollment, all patients in both studies received nivolumab, and the median duration of exposure was 110 (range: 15‐1635) days for patients with SQ NSCLC, with a median number of doses of 8 (range: 2‐114). For patients with non‐SQ NSCLC, median exposure was 74.5 (range: 1‐1716) days and the median number of doses was 6 (range: 1‐120). At the data cut‐off, two SQ NSCLC patients (5.7%) and six non‐SQ NSCLC patients (7.9%) continued to receive nivolumab, while 24 (68.6%) SQ NSCLC patients and 42 (55.3%) non‐SQ NSCLC patients received other systemic therapy after nivolumab in the primary studies (Table S1).

**Table 1:** Baseline characteristics of patients with squamous compared with non‐squamous non‐small cell lung cancer

	SQ (all patients) N = 35	Non‐SQ (all patients) N = 76
Age (y)		
Median (range)	65 (31‐85)	64 (39‐78)
≥65	20 (57.1)	36 (47.4)
Sex		
Male	32 (91.4)	49 (64.5)
Female	3 (8.6)	27 (35.5)
ECOG PS		
0	18 (51.4)	28 (36.8)
1	17 (48.6)	48 (63.2)
Brain metastasis		
Yes	3 (8.6)	21 (27.6)
Prior systemic regimens		
1	33 (94.3)	57 (75.0)
2	2 (5.7)	19 (25.0)
Smoking status		
Never	1 (2.9)	21 (27.6)
EGFR mutation status		
Positive	2 (5.7)	20 (26.3)
PD‐L1 expression level		
<1%	4 (21.1)	13 (32.5)
≥1%, <50%	10 (52.6)	20 (50.0)
≥50%	5 (26.3)	7 (17.5)

Note

Data are shown as n (%) unless otherwise specified.

Abbreviation: ECOG PS, Eastern Cooperative Oncology Group performance status; EGFR, epidermal growth factor receptor; PD‐L1, programmed death‐ligand 1; SQ, squamous.

### 3.2 Efficacy

**Figure 1:**
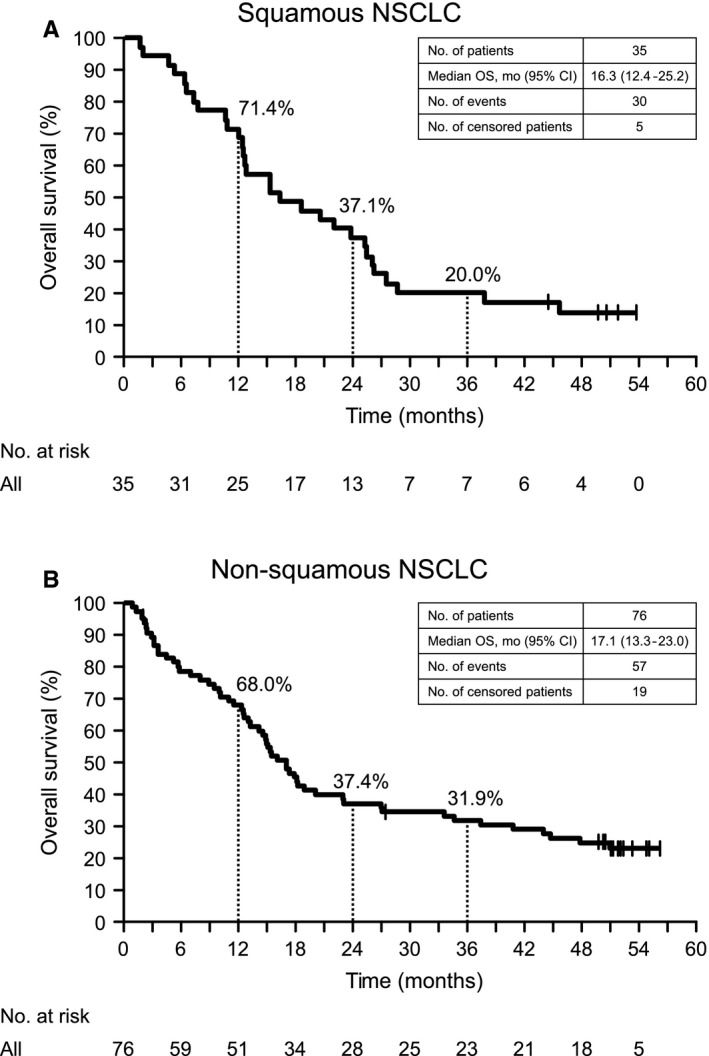
Kaplan–Meier curves of overall survival in patients with squamous (A) and non‐squamous (B) NSCLC at 3‐y follow‐up. NSCLC, non‐small cell lung cancer

In SQ NSCLC patients, the median OS was 16.3 months (95% CI: 12.4‐25.2); estimated 1‐, 2‐, and 3‐year survival rates were 71.4% (95% CI: 53.4‐83.5), 37.1% (95% CI: 21.6‐52.7), and 20.0% (95% CI: 8.8‐34.4), respectively; and seven patients (20%) were alive at year 3 (Table S2, Figure 1A). In non‐SQ NSCLC patients, the median OS was 17.1 months (95% CI: 13.3‐23.0); estimated 1‐, 2‐, and 3‐year survival rates were 68.0% (95% CI: 56.2‐77.3), 37.4% (95% CI: 26.5‐48.1), and 31.9% (95% CI: 21.7‐42.5), respectively (Table S2, Figure 1B). When SQ NSCLC and non‐SQ NSCLC patient data were pooled, the median OS was 17.1 months (95% CI: 14.2‐20.6), and the estimated 1‐, 2‐, and 3‐year survival rates were 69.1% (95% CI: 59.6‐76.8), 37.3% (95% CI: 28.3‐46.2), and 28.1% (95% CI: 20.0‐36.7), respectively (Table S3, Figure S1A).

**Figure 2:**
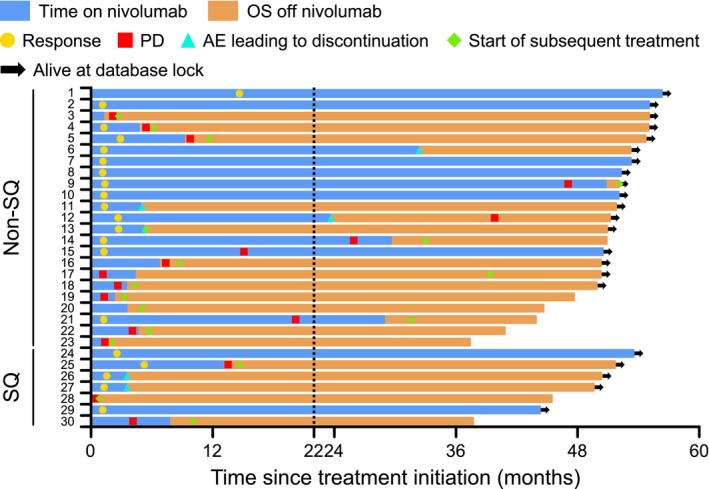
Duration of response among 3‐y survivors in non‐squamous and squamous NSCLC patients. NSCLC, non‐small cell lung cancer; OS, overall survival; PD, progressive disease; SQ, squamous

Five SQ NSCLC patients and 17 non‐SQ NSCLC patients were alive as of the data cut‐off date (Figure 2). Most 3‐year survivors lived for a prolonged period of time after discontinuation of nivolumab (Figure S2). Two SQ NSCLC patients and three non‐SQ NSCLC patients who discontinued nivolumab because of AEs lived for 3 years without subsequent systemic therapies. When the patient characteristics of 3‐year survivors and 3‐year nonsurvivors were compared, the proportions of patients who had received one prior therapy or had wild type EGFR status were significantly higher for 3‐year survivors (Table 2). The ORRs in 3‐year survivors and 3‐year nonsurvivors were 66.7% and 7.4%, respectively, and 20 of 26 (76.9%) responders survived ≥3 years (Figure S3A,B).

**Table 2:** Baseline characteristics of 3‐y survivors versus nonsurvivors for pooled squamous and non‐squamous non‐small cell lung cancer patients

	3‐y survivors N = 30	Non‐3‐y survivors N = 81	*P*‐value^a^
Median age (range)	63 (45‐71)	65 (31‐85)	.0992
Sex, male, %	76.7	71.6	.5938
ECOG PS 0, %	50.0	38.3	.2653
Brain metastasis, no, %	86.7	75.3	.1967
Prior systemic regimens, 1, %	93.3	76.5	.0449
Smoking status, former/current, %	86.7	77.8	.2968
EGFR mutation status, wild type/unknown, %	93.3	75.3	.0344
PD‐L1 expression level, %			.1588
<1%	17.6	33.3	
1%–<50%	52.9	50.0	
≥50%	29.4	16.7	

Abbreviation: ECOG PS, Eastern Cooperative Oncology Group performance status; EGFR, epidermal growth factor receptor; PD‐L1, programmed death‐ligand 1.

^a^ Wilcoxon's signed‐rank test was used for statistical comparisons of age and PD‐L1, and the chi‐square test was used for all other statistical comparisons.

**Figure 3:**
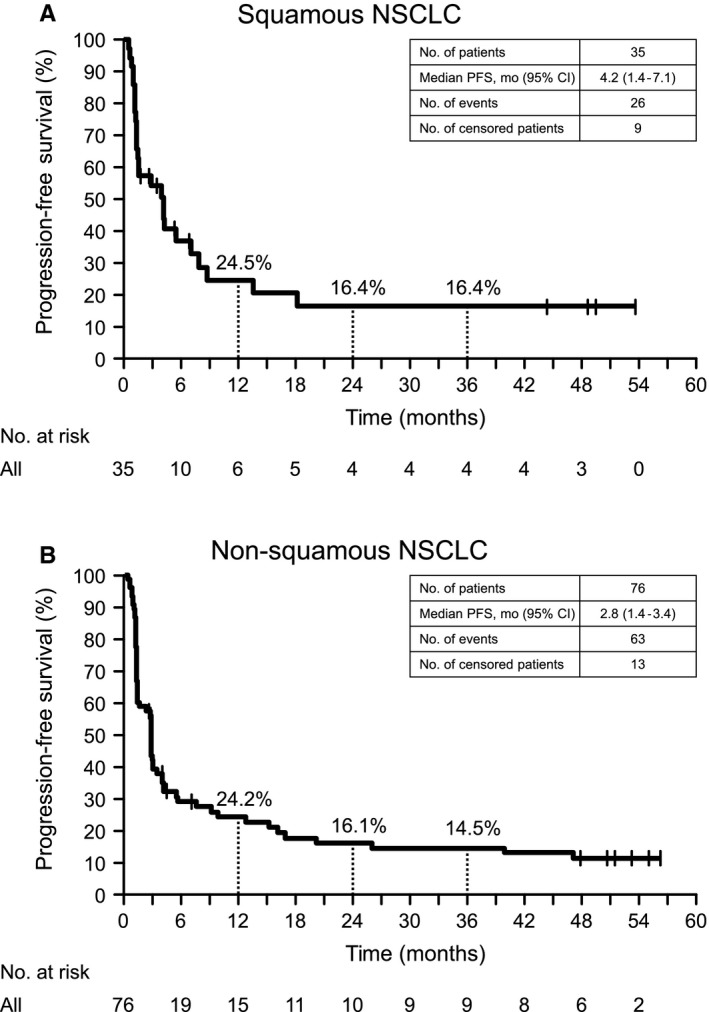
Kaplan–Meier curves of progression‐free survival in patients with squamous (A) and non‐squamous (B) NSCLC at 3‐y follow‐up. NSCLC, non‐small cell lung cancer

In SQ NSCLC patients, the median PFS (central assessment) was 4.2 months (95% CI: 1.4‐7.1); estimated 1‐, 2‐, and 3‐year PFS rates were 24.5% (95% CI: 10.7‐41.3), 16.4% (95% CI: 5.4‐32.5), and 16.4% (95% CI: 5.4‐32.5), respectively (Table S2); and four patients maintained PFS at year 3 (Figure 3A). In non‐SQ NSCLC patients, the median PFS (central assessment) was 2.8 months (95% CI: 1.4‐3.4); the estimated 1‐, 2‐, and 3‐year PFS rates were 24.2% (95% CI: 14.9‐34.7), 16.1% (95% CI: 8.5‐25.9), and 14.5% (95% CI: 7.3‐24.1), respectively; and nine patients maintained PFS at year 3 (Figure 3B). When SQ NSCLC and non‐SQ NSCLC patient data were pooled, the median PFS was 2.8 months (95% CI: 1.6‐4.0), and the estimated 1‐, 2‐, and 3‐year survival rates were 24.5% (95% CI: 16.5‐33.4), 16.3% (95% CI: 9.6‐24.5), and 15.2% (95% CI: 8.7‐23.2), respectively (Table S3 and Figure S1B).

In SQ NSCLC patients, the ORR (central assessment), DCR, and median DOR were 25.7% (95% CI: 14.2‐42.1), 54.3% (95% CI: 38.2‐69.5), and not reached (range: 3.0‐51.0 months), respectively (Table S2). In non‐SQ NSCLC patients, median ORR (central assessment), DCR, and DOR were 22.4% (95% CI: 14.5‐32.9), 47.4% (95% CI: 36.5‐58.4), and 41.5 months (range: 1.6‐53.8) (Table S2), respectively. When SQ NSCLC and non‐SQ NSCLC patient data were pooled, the median ORR (central assessment), DCR, and DOR were 23.4% (95% CI: 16.5‐32.1), 49.5% (95% CI: 40.4‐58.7), and 41.5 months (range: 1.6‐53.8), respectively (Table S3).

#### 3.2.1 Efficacy by PD‐L1 expression status

PD‐L1 expression status was available for 19 of 35 (54.3%) SQ NSCLC patients, and the proportions of patients with PD‐L1 expression <1%, 1%–<50%, and ≥50% were 21.1%, 52.6%, and 26.3%, respectively (Table 1). OS according to PD‐L1 expression was assessed by Kaplan‐Meier analysis (Figure S4A,B). The 3‐year survival rates for PD‐L1 expression <1%, ≥1%, 1%–<50%, and ≥50% were 0.0%, 20.0%, 20.0%, and 20.0%, respectively (Table S2).

For non‐SQ NSCLC patients, PD‐L1 expression status was available for 40 of 76 (88.9%) patients, and the proportions of patients with PD‐L1 expression <1%, 1%–<50%, and ≥50% were 32.5%, 50.0%, and 17.5%, respectively (Table 1). The corresponding OS Kaplan–Meier curves are shown in Figure S4C,D. The 3‐year survival rates for PD‐L1 expression <1%, ≥1%, 1%–<50%, and ≥50% were 23.1%, 44.1%, 39.4%, and 57.1%, respectively (Table S2). Three‐year survivors were observed among non‐SQ NSCLC patients, regardless of PD‐L1 expression. PFS according to PD‐L1 expression (Kaplan‐Meier analysis) is shown in Figure S5A‐D.

### 3.3 Safety

In both groups, treatment‐related AEs were primarily grades 1‐2, and the overall pattern and frequency of the most common AEs (ie, observed in ≥10% of patients) did not change with longer nivolumab treatment. In SQ NSCLC patients, the frequencies of treatment‐related AEs of all grades and grades 3‐4 were 68.6% (n = 24) and 8.6% (n = 3), respectively; the frequency of treatment‐related AEs leading to withdrawal was 8.6% (n = 3); and no treatment‐related deaths occurred (Table 3). In non‐SQ NSCLC patients, the frequencies of treatment‐related AEs of all grades and grades 3‐4 were 86.8% (n = 66) and 23.7% (n = 18), respectively; the frequency of treatment‐related AEs leading to withdrawal was 17.1% (n = 13); and no treatment‐related deaths occurred (Table 3).

**Table 3:** Safety summary in separate and pooled squamous and non‐squamous non‐small cell lung cancer patients

AE	SQ N = 35	Non‐SQ N = 76	Non‐SQ + SQ N = 111
Treatment‐related AE (all grades)	24 (68.6)	66 (86.8)	90 (81.1)
Treatment‐related AE (grades 3‐4)	3 (8.6)	18 (23.7)	21 (18.9)
Treatment‐related serious AE	2 (5.7)	16 (21.1)	18 (16.2)
Treatment‐related AE leading to discontinuation	3 (8.6)	13 (17.1)	16 (14.4)

Note

Data are shown as n (%).

AE, adverse event; SQ, squamous.

**Figure 4:**
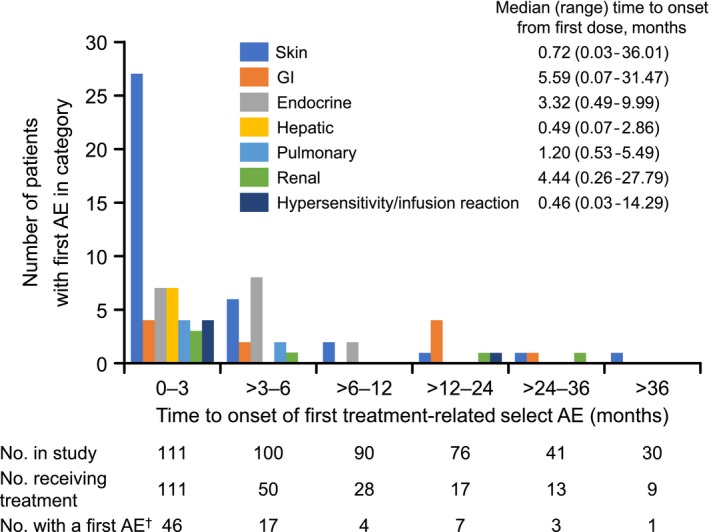
Reported time to onset of first treatment‐related selected AEs in pooled squamous and non‐squamous NSCLC patients. ^†^Patients with one or more selected AEs in a given category were counted only once in the time interval corresponding to the first event; patients with multiple events from different categories within the same time interval were counted once in each category. AE, adverse event; GI, gastrointestinal; NSCLC, non‐small cell lung cancer

When SQ NSCLC and non‐SQ NSCLC patient data were pooled, the frequencies of treatment‐related AEs of all grades and grades 3‐4 were 81.1% and 18.9%, respectively (Table 3). The main treatment‐related AEs were decreased appetite (14.4%), malaise (14.4%), pyrexia (14.4%), rash (14.4%), and nausea (10.8%) (Table 4). The only grade 3 or 4 treatment‐related AE with a frequency ≥ 2% was lymphocyte count decreased (4.5%). The majority of treatment‐related selected AEs occurred within the first 3 months of nivolumab treatment (Figure 4). A total of 44 new treatment‐related AEs occurred from 1 year after initiation of nivolumab, and grade ≥3 events were hypophosphatemia (grade 3, SQ), enterocolitis (grade 3, non‐SQ) and amylase increased (grade 3, non‐SQ) (Table S4). Of note, no new onset of pneumonitis or interstitial lung disease was reported from 1 year after initiation of nivolumab onward.

**Table 4:** Separate and pooled analyses (squamous and non‐squamous NSCLC patients) of the frequency of treatment‐related AEs (≥5% of patients)

	ONO‐4538‐05 Study N = 35		ONO‐4538‐06 Study N = 76		Total (N = 111)	
	All grades	Grades 3‐4	All grades	Grades 3‐4	All grades	Grades 3‐4
Any AE	24 (68.6)	3 (8.6)	66 (86.8)	18 (23.7)	90 (81.1)	21 (18.9)
Decreased appetite	5 (14.3)	0 (0.0)	11 (14.5)	1 (1.3)	16 (14.4)	1 (0.9)
Malaise	5 (14.3)	0 (0.0)	11 (14.5)	0 (0.0)	16 (14.4)	0 (0.0)
Pyrexia	5 (14.3)	0 (0.0)	11 (14.5)	0 (0.0)	16 (14.4)	0 (0.0)
Rash	5 (14.3)	0 (0.0)	11 (14.5)	0 (0.0)	16 (14.4)	0 (0.0)
Nausea	3 (8.6)	0 (0.0)	9 (11.8)	0 (0.0)	12 (10.8)	0 (0.0)
Fatigue	1 (2.9)	0 (0.0)	9 (11.8)	1 (1.3)	10 (9.0)	1 (0.9)
Pruritus	1 (2.9)	0 (0.0)	9 (11.8)	1 (1.3)	10 (9.0)	1 (0.9)
Lymphocyte count decreased	3 (8.6)	2 (5.7)	7 (9.2)	3 (3.9)	10 (9.0)	5 (4.5)
Diarrhea	3 (8.6)	0 (0.0)	6 (7.9)	0 (0.0)	9 (8.1)	0 (0.0)
Hypothyroidism	0 (0.0)	0 (0.0)	7 (9.2)	0 (0.0)	7 (6.3)	0 (0.0)
Constipation	1 (2.9)	0 (0.0)	6 (7.9)	0 (0.0)	7 (6.3)	0 (0.0)
Rash maculopapular	2 (5.7)	0 (0.0)	5 (6.6)	0 (0.0)	7 (6.3)	0 (0.0)
Arthralgia	2 (5.7)	0 (0.0)	5 (6.6)	0 (0.0)	7 (6.3)	0 (0.0)
Acneiform eruption	2 (5.7)	0 (0.0)	4 (5.3)	0 (0.0)	6 (5.4)	0 (0.0)

Note

Data are shown as n (%).

No grade 5 treatment‐related adverse events were reported.

AE, adverse event; SQ, squamous; NSCLC, non‐small cell lung cancer.

#### 3.3.1 Treatment‐related selected AEs and efficacy

**Figure 5:**
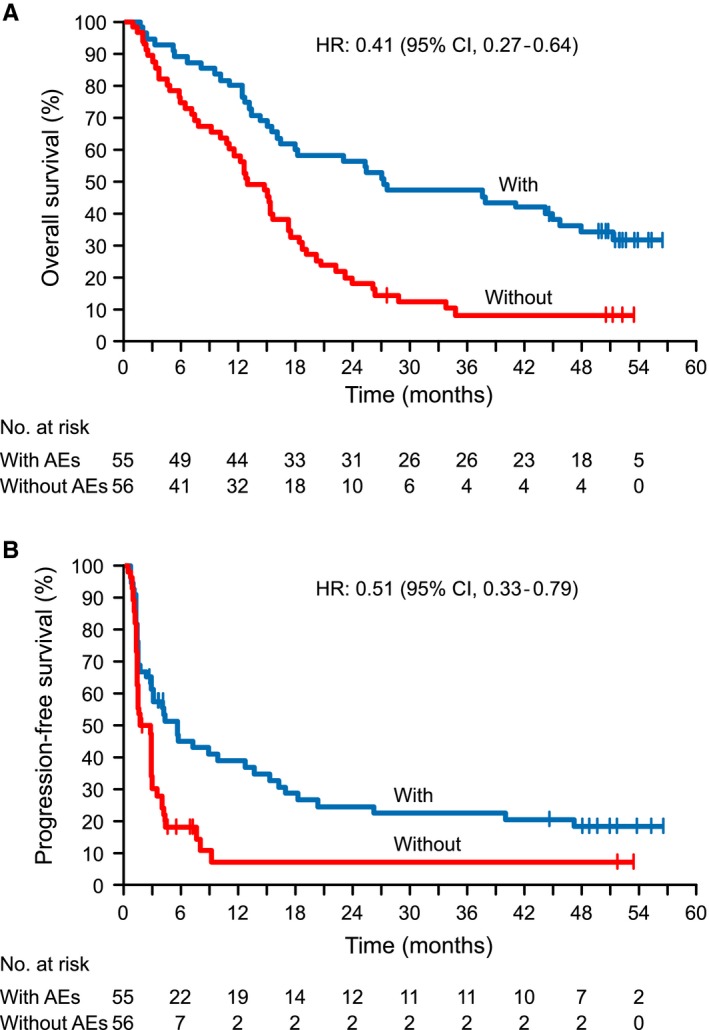
Association between treatment‐related selected AE incidence and (A) overall survival and (B) progression‐free survival in pooled squamous and non‐squamous NSCLC patients. AE, adverse event; NSCLC, non‐small cell lung cancer; HR, hazard ratio; CI, confidence interval

Higher ORRs were observed in patients with treatment‐related selected AEs, regardless of the severity (Table S5), and PFS and OS were longer in patients with a treatment‐related selected AE than those without (Figure 5A,B). A 6‐week landmark analysis also showed that the ORR was higher, and OS was longer in patients with treatment‐related selected AEs than in those without (Table S5). When treatment‐related AEs were analyzed by category, the ORR, 6‐month PFS rate, and 1‐year survival rate showed greater improvements in patients with, than in those without, rash, endocrine disorders, and gastrointestinal toxicity (Table 5).

**Table 5:** OS, 6‐mo PFS, and 1‐year ORR by treatment‐related AE in pooled squamous and non‐squamous non‐small cell lung cancer patients

	Treatment‐related selected AE	n	ORR, %		6‐mo PFS rate, %		1‐y OS rate, %	
Pyrexia	＋	16	31.3	*P* = .4243	42.2	*P* = .3131	87.5	*P* = .1023
	−	95	22.1		29.7		66.0	
Rash	＋	16	43.8	*P* = .0380	42.2	*P* = .0503	87.5	*P* = .0008
	−	95	20.0		29.5		66.0	
Endocrine disorders	＋	16	50.0	*P* = .0067	56.3	*P* = .0529	87.5	*P* = .0162
	−	95	18.9		26.8		66.0	
Pulmonary toxicity	＋	6	33.3	*P* = .5557	44.4	*P* = .2427	50.0	*P* = .8847
	−	105	22.9		30.7		70.2	
Gastrointestinal toxicity	＋	11	63.6	*P* = .0009	80.8	*P* = .0167	72.7	*P* = .0298
	−	100	19.0		26.0		68.7	
Hepatotoxicity	＋	7	14.3	*P* = .5554	14.3	*P* = .2837	0.0	*P* = .3412
	−	104	24.0		32.8		67.0	

Abreviation: AE, adverse event; ORR, overall response rate; OS, overall survival; PFS, progression‐free survival.

When the characteristics of patients with and without treatment‐related selected AEs were compared, the proportions of patients with ECOG PS 0, who had never smoked, and with positive EGFR mutation status were lower for those who reported treatment‐related selected AEs (Table S6). The median numbers of doses of nivolumab among patients with and without treatment‐related selected AEs were 9 and 6, respectively (Table S6).

### 3.4 Efficacy and safety by antinuclear antibody status

ORR, PFS, and OS by antinuclear antibody status are shown in Table S8. ORR, PFS, and OS were all comparable between patients positive and negative for baseline blood antinuclear antibodies. Incidence of treatment‐related AEs of all grades and grades 3‐4, and the frequency of serious treatment‐related AEs by antinuclear antibody status are shown in Tables S7 and S8. The profiles of treatment‐related AEs were similar between patients positive and negative for antinuclear antibodies.

## 4 DISCUSSION

In this analysis of the ONO‐4538‐05 and ONO‐4538‐06 studies, the continued clinical efficacy of nivolumab in Japanese patients with either SQ or non‐SQ NSCLC noted in the primary phase II analysis of this study was reinforced. The safety profile was also found to be acceptable, consistent with that observed in the individual primary analyses.^9^, ^10^

The median duration of response to nivolumab was ≥3 years, demonstrating its long‐term effectiveness. Three‐year survival rates in this study were comparable with those reported in previous phase III studies of nivolumab. There were some 3‐year survivors among the patients with either stable or progressive disease, but the majority were responders, of whom 76.9% survived ≥3 years. In a previous report of pooled phase III studies (CheckMate 017/057) of nivolumab, 46 out of 83 (55.4%) nivolumab responders and 13 out of 48 (27.1%) docetaxel responders survived ≥3 years.^14^ Taken together, these results suggest that the possibility of long‐term survival increases in patients responding to nivolumab compared with those receiving conventional chemotherapy.

The proportion of patients receiving one prior therapy or having wild type EGFR status was high among the 3‐year survivors. Given that patients with an EGFR mutation typically have a history of treatment with EGFR tyrosine kinase inhibitors, a relationship may exist between EGFR mutation status and number of prior therapies. Nivolumab efficacy was higher in patients who reported a treatment‐related selected AE (irAE) as a result of nivolumab treatment compared with those who did not report an irAE. The number of doses of nivolumab was higher in patients with an irAE than in those without, so the duration of nivolumab exposure may potentially have affected this outcome.

A 6‐week landmark analysis, performed to exclude early progressors, showed that nivolumab efficacy was higher in patients who reported an irAE compared with those who did not, although the differences decreased. The occurrence of nivolumab‐induced irAEs might therefore correlate with efficacy, even disregarding the effect of the duration of exposure. When the relationship between the occurrence of nivolumab‐induced irAEs and nivolumab efficacy was analyzed by irAE category, the occurrence of rash, endocrine disorders, and gastrointestinal toxicity was correlated with the ORR to nivolumab. The occurrence of these three toxicities has previously been correlated with the efficacy of anti‐PD‐1 antibodies,^15^, ^16^, ^17^, ^18^, ^19^ and the present data appear to support these findings.

Baseline antinuclear antibody positivity did not affect the safety and efficacy of nivolumab. The frequency of irAEs has previously been shown to increase in patients with a history of autoimmune disease who were administered with an immune checkpoint inhibitor. Furthermore, the frequency of anti‐PD‐1 antibody‐induced thyroid dysfunction appears to increase in the presence of baseline thyroid‐related autoantibody positivity.^20^, ^21^ A relationship between antinuclear antibodies and immune checkpoint inhibitors with respect to safety has not been reported to date, and the present data indicate that these factors are not correlated. A substantial proportion of patients were found to express PD‐L1 in the present study, and previous data have suggested that these patients might be at increased risk of a poorer prognosis.^6^ Consequently, it was deemed important to assess the effects of nivolumab in the subset of patients expressing PD‐L1. The pooled results of efficacy by PD‐L1 expression in both SQ and non‐SQ NSCLC patients were consistent with those of the individual reports (ONO‐4538‐05, ONO‐4538‐06).^9^, ^10^ The 3‐year OS and PFS rates showed that, after 3 years of follow‐up, nivolumab continued to demonstrate long‐term efficacy in previously treated patients with SQ or non‐SQ NSCLC. No new safety signals were identified for nivolumab after 3 years of follow‐up, and nivolumab maintained a favorable safety profile.

This updated analysis has some limitations, including the inclusion of only Japanese patients, the lack of heterogeneity, the relatively small sample size, and the absence of a comparator in both studies’ patient populations.

In conclusion, long‐term collective analyses from the ONO‐4538‐05 and ONO‐4538‐06 studies continue to support the sustained safety and clinical efficacy of nivolumab and provide evidence to support the ability of nivolumab to improve OS in Japanese patients with previously treated advanced SQ and non‐SQ NSCLC. Future research will investigate approaches to increase the proportion of patients experiencing long‐term survival, including combination studies with other immune checkpoint inhibitors or chemotherapies and the identification of specific biomarkers capable of predicting the response to these therapies.

## CONFLICT OF INTEREST

Hidehito Horinouchi received a grant from the study sponsor, related to the current research; and grants from Astellas, Merck Serono, and Genomic Health; personal fees from Lilly, and grants and personal fees from BMS, Novartis, Taiho, Chugai, AstraZeneca, and MSD, outside the submitted work. Makoto Nishio received a grant and personal fees from the study sponsor, related to the current research; and grants from F Hoffmann‐La Roche and Astellas; personal fees from Boehringer‐Ingelheim, Sankyo Healthcare, Taiho Pharmaceutical, and Merck Serono; and grants and personal fees from Bristol‐Myers Squibb, Pfizer, Chugai Pharmaceutical, Eli Lilly, Taiho Pharmaceutical, AstraZeneca, MSD, and Novartis, outside the submitted work. Toyoaki Hida received a grant and personal fees from the study sponsor, related to the current research; and grants from Merck Serono, Eisai, Takeda Pharmaceutical Co., Ltd., Dainippon Sumitomo Pharma, AbbVie, Kyowa Hakko Kirin, Daiichi Sankyo, Astellas, and Servier; and grants and personal fees from Ono Pharmaceutical Co., Ltd., Bristol‐Myers Squibb, Chugai Pharmaceutical Co., Ltd., AstraZeneca, Nippon Boehringer Ingelheim, Novartis, Eli Lilly, Taiho Pharmaceutical Co., Ltd., Pfizer, Clovis Oncology, MSD, Kissei, and Ignyta, outside the submitted work. Kazuhiko Nakagawa received a grant from Merck Serono Co., Ltd. and grants and personal fees from the study sponsor, Bristol‐Myers Squibb Company, MSD KK, Chugai Pharmaceutical Co., Ltd. and AstraZeneca, related to the current research; and grants from A2 Healthcare Corp, inVentiv Health Japan, Daiichi Sankyo Co., Ltd., AbbVie Inc, Quintiles Inc, ICON Japan KK, Takeda Pharmaceutical Co., Ltd., EP‐CRSU Co., Ltd., Gritstone Oncology, Inc, Linical Co., Ltd., Eisai Co., Ltd., Parexel International Corp., EPS International Co., Ltd., Yakult Honsha Co., Ltd., Otsuka Pharmaceutical Co., Ltd., AC Medical Inc, Japan Clinical Research Operations, PPD‐SNBL KK, and Covance Inc; personal fees from Clinical Trial Co., Ltd., Nippon Boehringer Ingelheim Co., Ltd., SymBio Pharmaceuticals Ltd., Pfizer Japan Inc, EPS Holdings Inc, Showa Yakuhin Kako Co., Ltd., Ayumi Pharmaceutical Corporation and Kissei Pharmaceutical Co., Ltd.; and grants and personal fees from Astellas Pharma Inc, Novartis Pharma KK, Eli Lilly Japan KK, Taiho Pharmaceutical Co., Ltd., and Kyowa Hakko Kirin, outside the submitted work. Hiroshi Sakai received personal fees from the study sponsor, related to the current research; and personal fees from Chugai Pharma, AstraZeneca, and Bristol‐Myers Squibb, outside the submitted work. Naoyuki Nogami received a grant and personal fees from the study sponsor, related to the current research; and personal fees from Pfizer Inc, Kyowa Hakko Kirin, Taiho Pharmaceutical Co., Ltd., Chugai Pharmaceutical Co., Ltd., Eli Lilly Japan, Boehringer Ingelheim, MSD, AstraZeneca, and Bristol‐Myers Squibb, outside the submitted work. Shinji Atagi received a grant and personal fees from the study sponsor, related to the current research; and personal fees from Hisamitsu and grants and personal fees from MSD, Chugai, AstraZeneca, Taiho, Boehringer Ingelheim, Pfizer, and Bristol‐Myers Squibb, outside the submitted work. Toshiaki Takahashi received a grant and personal fees from the study sponsor, related to the current research; and a grant from Pfizer Japan Inc.; personal fees from Boehringer Ingelheim Japan Inc, and Roche Diagnostics KK; and grants and personal fees from MSD KK, AstraZeneca KK, Chugai Pharmaceutical Co., Ltd., and Eli Lilly Japan KK, outside the submitted work. Hideo Saka received a grant from the study sponsor, related to the current research; and grants from AstraZeneca KK, MSD KK, Bristol‐Myers Squibb KK, Chugai Pharmaceutical Co., Ltd., and Eli Lilly Japan KK, outside the submitted work. Mitsuhiro Takenoyama received a grant from the study sponsor, related to the current research; and grants from Johnson & Johnson, Kaketsuken, Novartis Pharma KK, and Yakult Honsha Co., Ltd., personal fees from MSD KK; and grants and personal fees from AstraZeneca KK, Bristol‐Myers Squibb KK, Chugai Pharmaceutical Co., Ltd., Covidien Japan KK, Eli Lilly Japan, KK, Kyowa Hakko Kirin Co., Ltd., Nippon Boehringer Ingelheim Co., Ltd., Ono Pharmaceutical Co., Ltd., and Taiho Pharmaceutical Co., Ltd., outside the submitted work. Nobuyuki Katakami received a grant from the study sponsor, related to the current research; and personal fees from Amgen, Boehringer Ingelheim Pharma, Chugai Pharma, Eli Lilly Pharma, Novartis Pharma KK, Ono Pharma, Pfizer, Taiho Pharma, MSD, and Daiichi Sankyo, outside the submitted work. Hiroshi Tanaka received a grant and personal fees from the study sponsor, related to the current research; and a grant from Merck Serono; personal fees from Novartis; and grants and personal fees from Bristol‐Myers Squibb, Eli Lilly, MSD, Taiho Pharmaceutical, Pfizer, Chugai Pharmaceutical, AstraZeneca, and Boehringer Ingelheim, outside the submitted work. Koji Takeda received a grant from the study sponsor, related to the current research; and grants from Merck Serono and MSD; personal fees from Daiichi Sankyo Co., Ltd., Kyowa Hakko Kirin Co., Ltd., and Novartis; and grants and personal fees from AstraZeneca, Boehringer Ingelheim, Bristol‐Myers Squibb, Chugai Pharmaceutical Co., Ltd., Eli Lilly, Ono Pharmaceutical Co., Ltd., Pfizer, and Taiho Pharmaceutical Co., Ltd., outside the submitted work. Miyako Satouchi received a grant and personal fees from the study sponsor, related to the current research; and grants from Ignyta, AbbVie, and Takeda; personal fees from Taiho Pharmaceutical; and grants and personal fees from Bristol‐Myers Squibb, Chugai Pharmaceutical, Eli Lilly Japan, Pfizer Japan, AstraZeneca, MSD, Boehringer Ingelheim, and Novartis Pharmaceutical, outside the submitted work. Hiroshi Isobe received personal fees from AstraZeneca KK, Bristol‐Myers Squibb Co., Ono Pharmaceutical Co., and Taiho Pharmaceutical Co., outside the submitted work. Makoto Maemondo received personal fees from Ono Pharma, MSD, and AstraZeneca; and a grant and personal fees from Chugai, outside the submitted work. Koichi Goto received grants and personal fees from the study sponsor and Bristol‐Myers Squibb, related to the current research; and grants from Ignyta, OxOnc, Sumitomo Dainippon Pharma, Astellas Pharm, Amgen Astellas BioPharma, Eisai, Kyowa Hakko Kirin, Janssen Pharmaceutical, and RTI Health Solutions; personal fees from F Hoffmann‐La Roche, Otsuka Pharmaceutical, SRL, and Nippon Kayaku; and grants and personal fees from Eli Lilly, AbbVie Stemcentrx, Life Technologies Japan, Riken Genesis, AstraZeneca, Boehringer Ingelheim, Chugai Pharmaceutical, Daiichi Sankyo, MSD, Merck Serono, Novartis Pharma, Pfizer, Taiho, and Takeda Pharmaceutical, outside the submitted work. Tomonori Hirashima received a grant from the study sponsor, related to the current research; and grants from Chugai, Taiho, MSD, AstraZeneca, Lilly Japan, Merck Serono, Bristol‐Myers Squibb, and Boehringer Ingelheim, outside the submitted work. Koichi Minato received a grant from the study sponsor, related to the current research; and grants from AstraZeneca and Bristol‐Myers Squibb, outside the submitted work. Naoki Sumiyoshi is an employee of the study sponsor. Tomohide Tamura received personal fees from Eli Lilly, Ono, Chugai, Taiho, Boehringer Ingelheim, Bristol‐Myers Squibb, MSD, Kyowa Kirin, and ShiftZero, outside the submitted work.

## Data Availability

At this time, individual participant data have not been made available; however, reasonable requests made to the corresponding author for de‐identified patient‐level data that underlie the results reported in this article will be considered.
