# Supplementary material for: Three‐year follow‐up results from phase II studies of nivolumab in Japanese patients with previously treated advanced non‐small cell lung cancer: Pooled analysis of ONO‐4538‐05 and ONO‐4538‐06 studies
Source: Cancer Med. 2019 Jul 29;8(11):5183–93. doi: 10.1002/cam4.2411 (PMC6718542; doi:10.1002/cam4.2411)
Supplement: Supplementary file 1 [file CAM4-8-5183-s001.pdf]

Figure S1. Kaplan–Meier curves of (A) overall survival and (B) progression-free survival in pooled squamous and non-squamous NSCLC populations.

NSCLC, non-small cell lung cancer

A

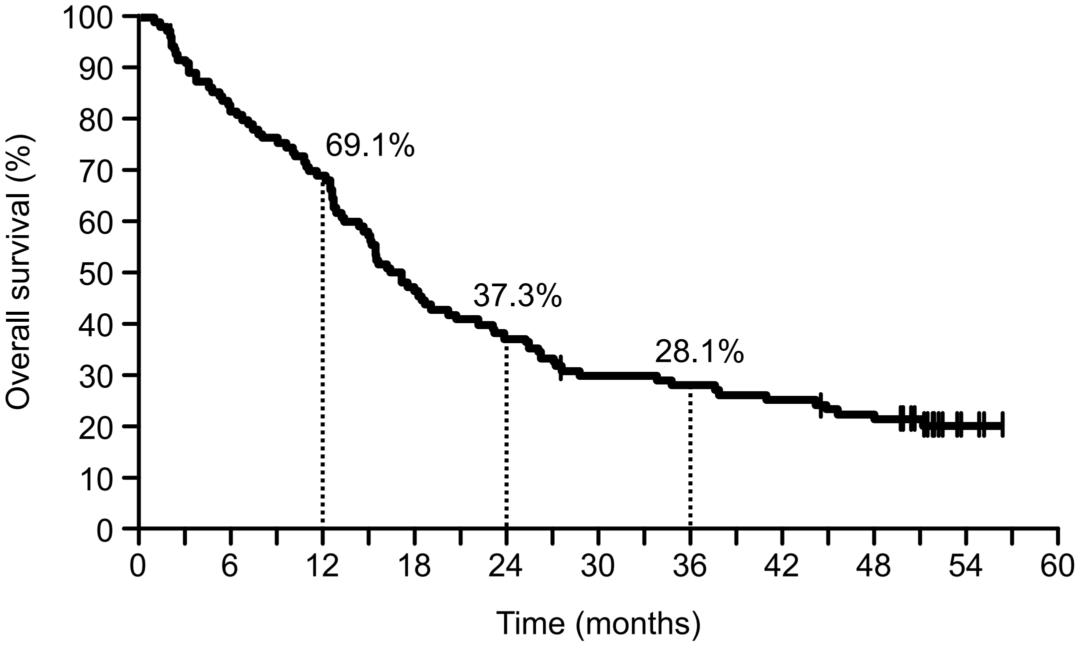

No. at risk

All	111	90	76	51	41	32	30	27	22	5

Figure S1

B

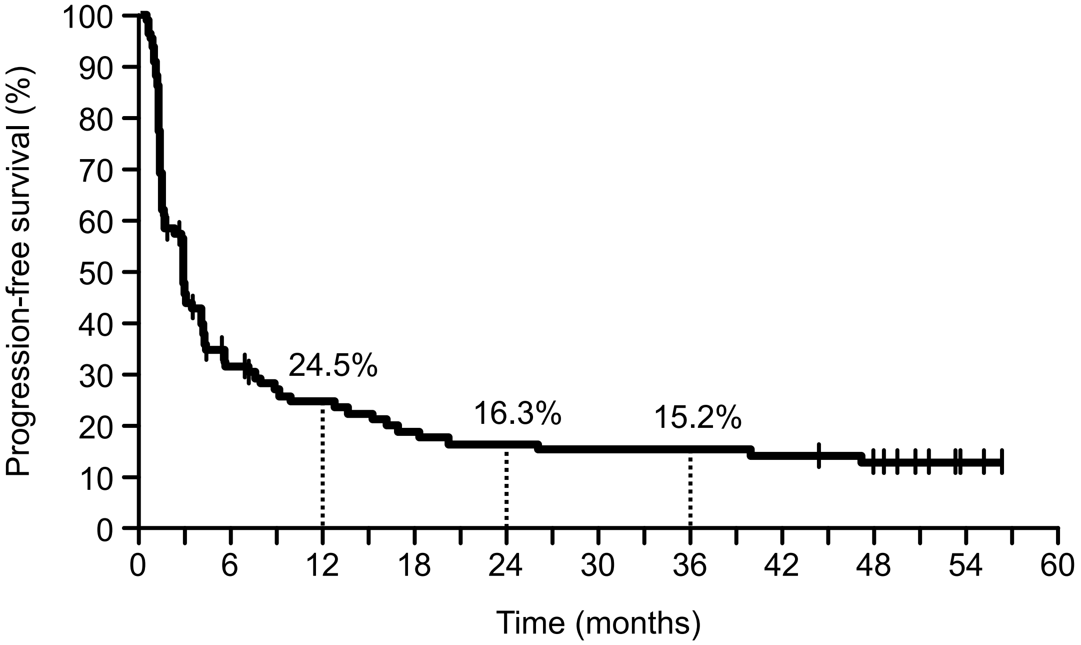

No. at risk

All	111	29	21	16	14	13	13	12	9	2

Figure S2. Patients who discontinued following an AE.

AE, adverse event; OS, overall survival; PD, progressive disease; SQ, squamous.

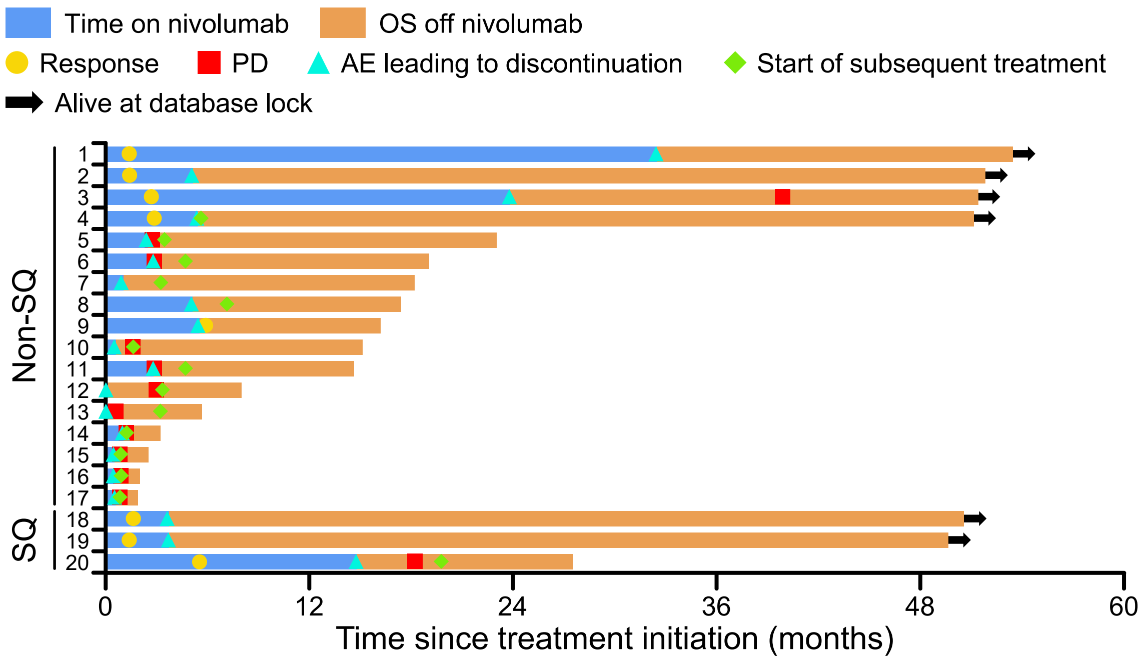

Figure S3. Patients with (A) SQ NSCLC and (B) non-SQ NSCLC by best overall response time.

AE, adverse event; CR, complete response; NE, not evaluable; NSCLC, non-small cell lung cancer; OS, overall survival; PD, progressive disease; PR, partial response; SD, stable disease; SQ, squamous.

A

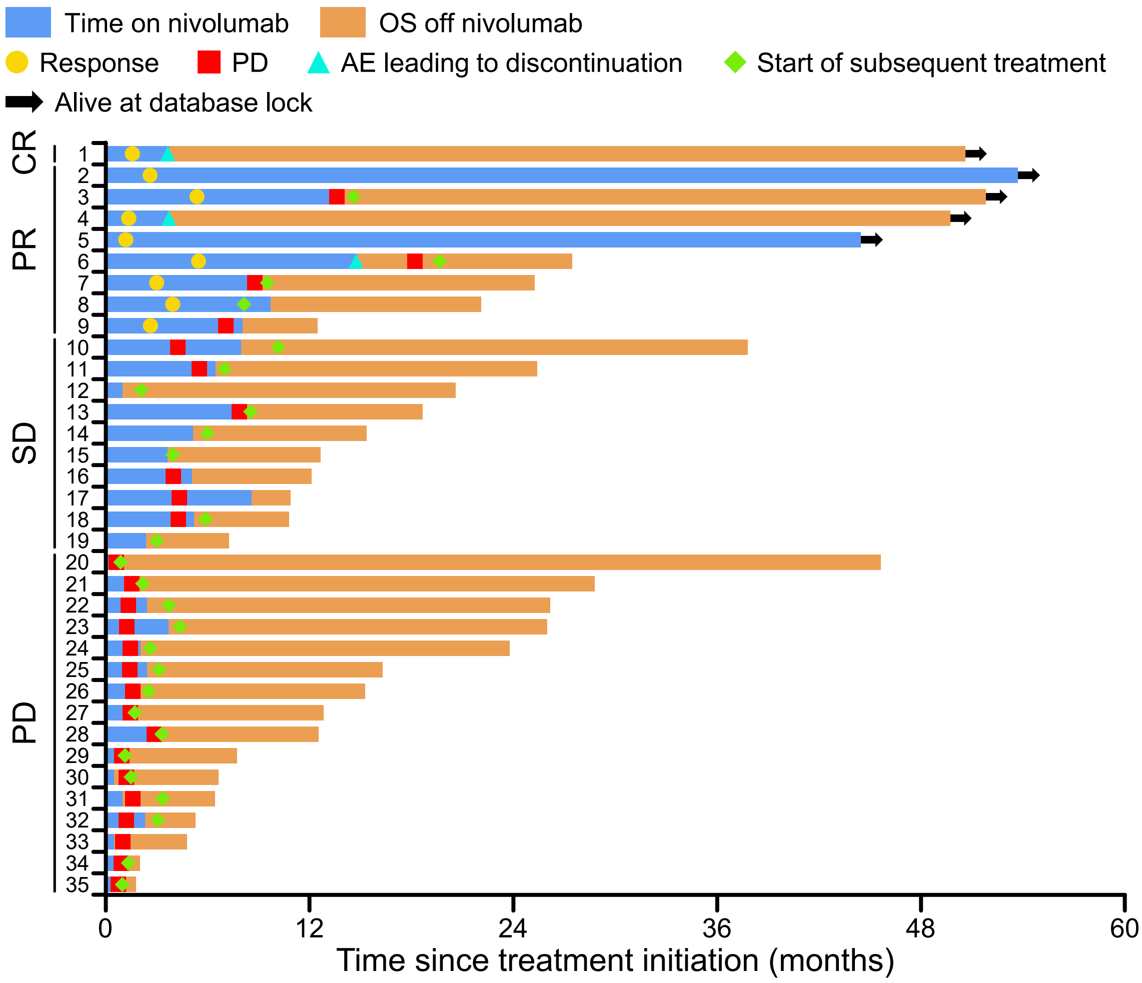

Figure S3

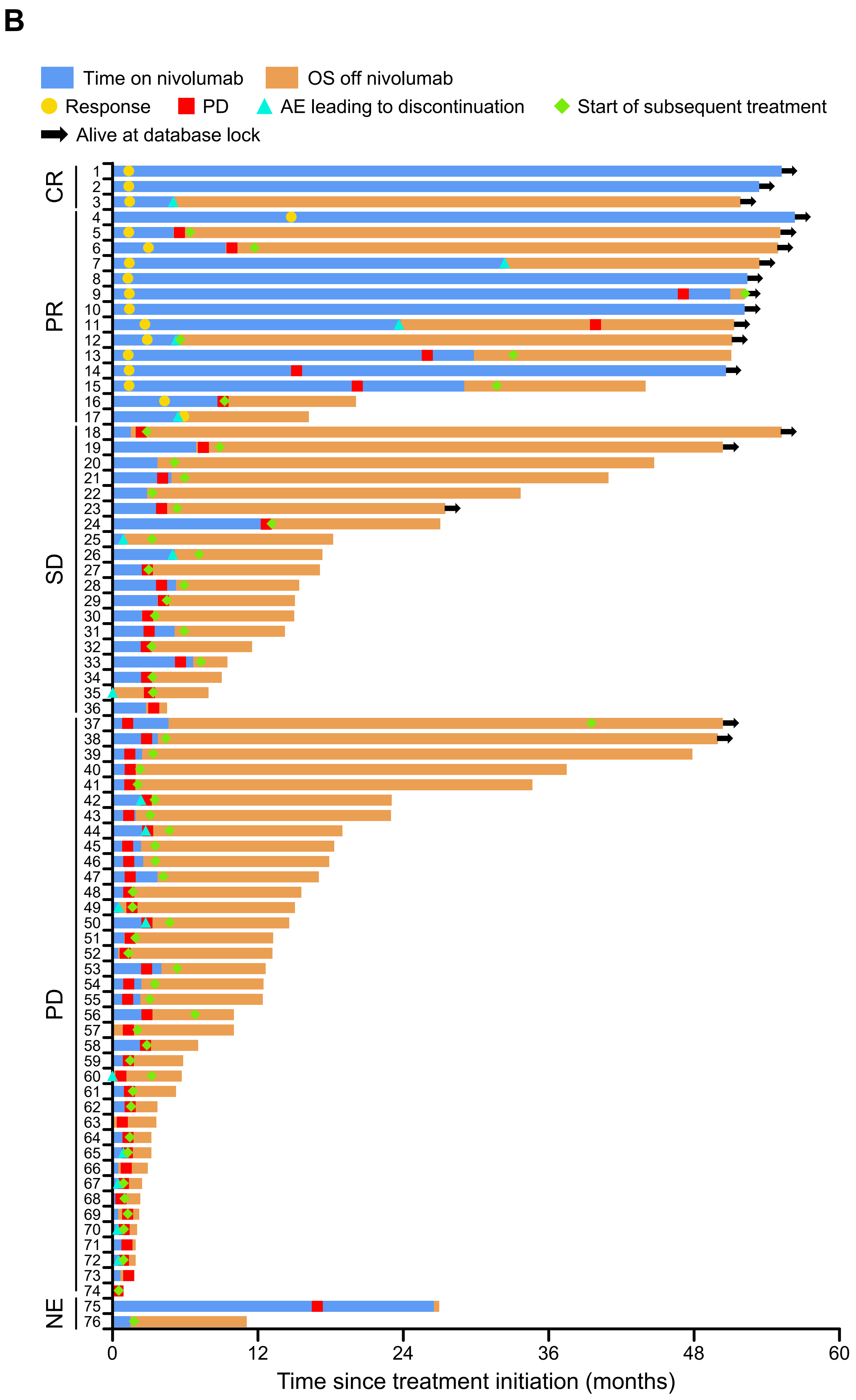
B
Time on nivolumab
OS off nivolumab
Response
PD
AE leading to discontinuation
Start of subsequent treatment
Alive at database lock
CR
PR
SD
PD
NE
0
12
24
36
48
60
Time since treatment initiation (months)

Figure S4. Kaplan–Meier curves of overall survival at 3-year follow-up stratified by PD-L1 expression. (A) squamous NSCLC by PD-L1 expression <1% and ≥1%, (B) squamous NSCLC by PD-L1 expression <1%, ≥1% to <50%, and ≥50%, (C) non-squamous NSCLC by PD-L1 expression <1% and ≥1%, (D) non-squamous NSCLC by PD-L1 expression <1%, ≥1% to <50%, and ≥50%.
PD-L1, programmed death-ligand 1; NSCLC, non-small cell lung cancer.

A

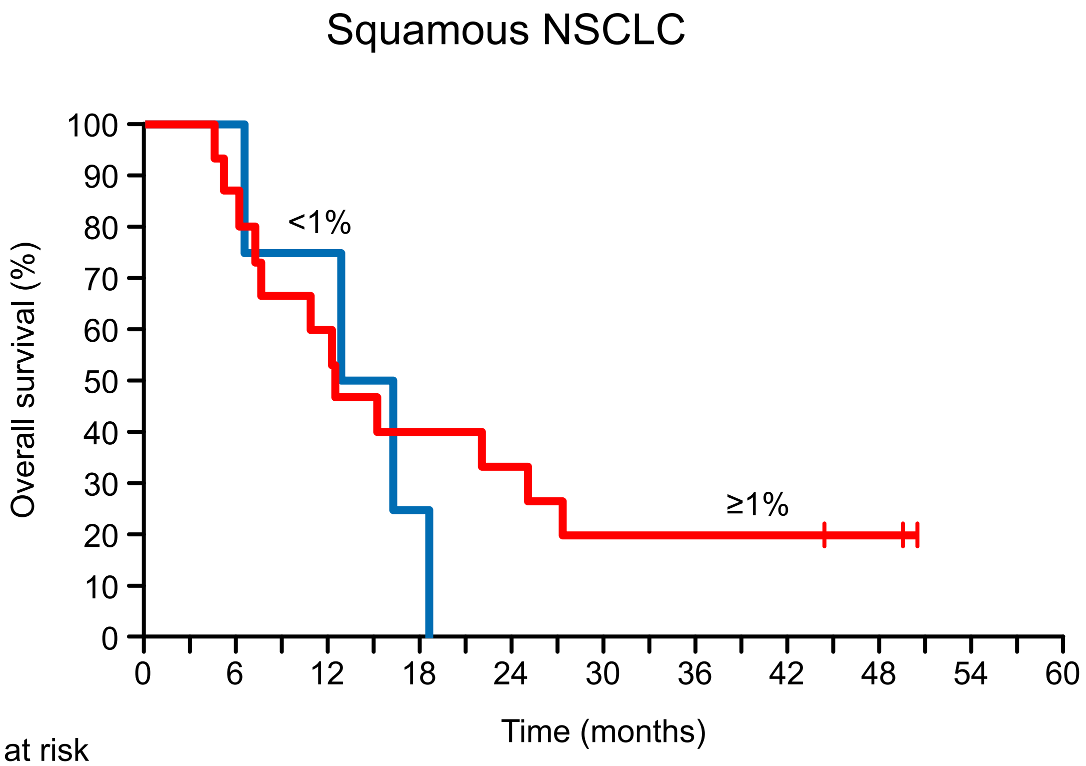

No. at risk
PD-L1

<1%	4	4	3	1	0	0	0	0	0	0
≥1%	15	13	9	6	5	3	3	3	2	0

Figure S4

B

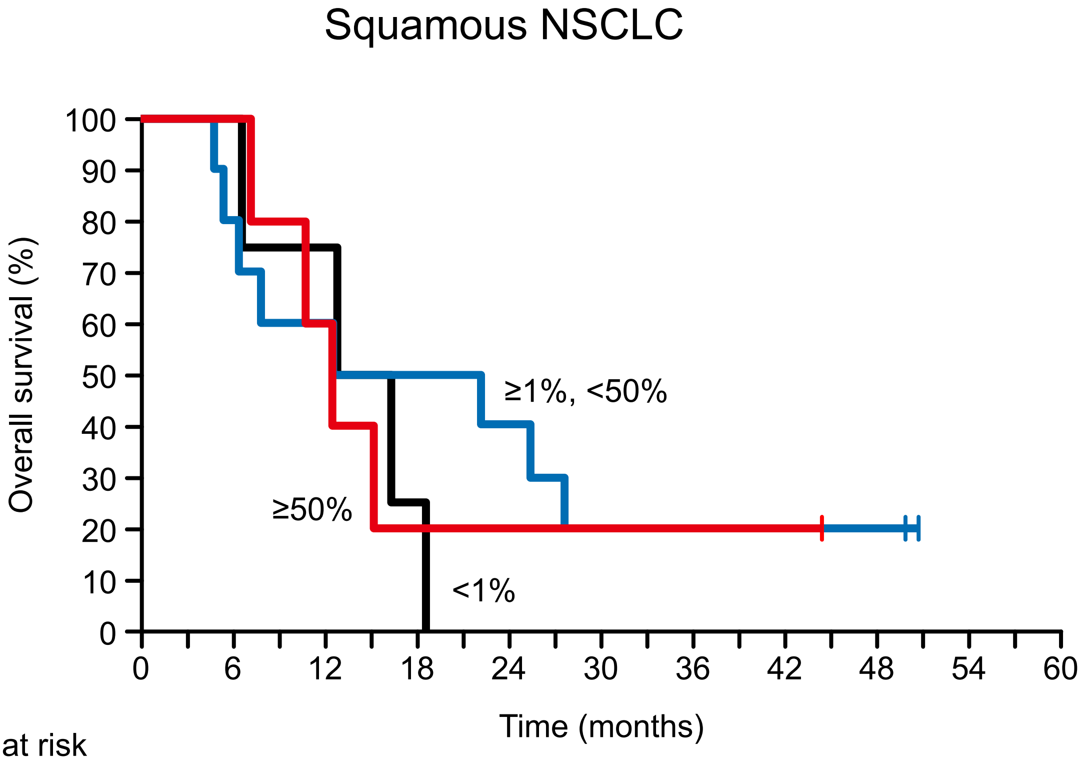

No. at risk

PD-L1										
<1%	4	4	3	1	0	0	0	0	0	0
≥1%, <50%	10	8	6	5	4	2	2	2	2	0
≥50%	5	5	3	1	1	1	1	1	0	0

Figure S4

C

Non-squamous NSCLC

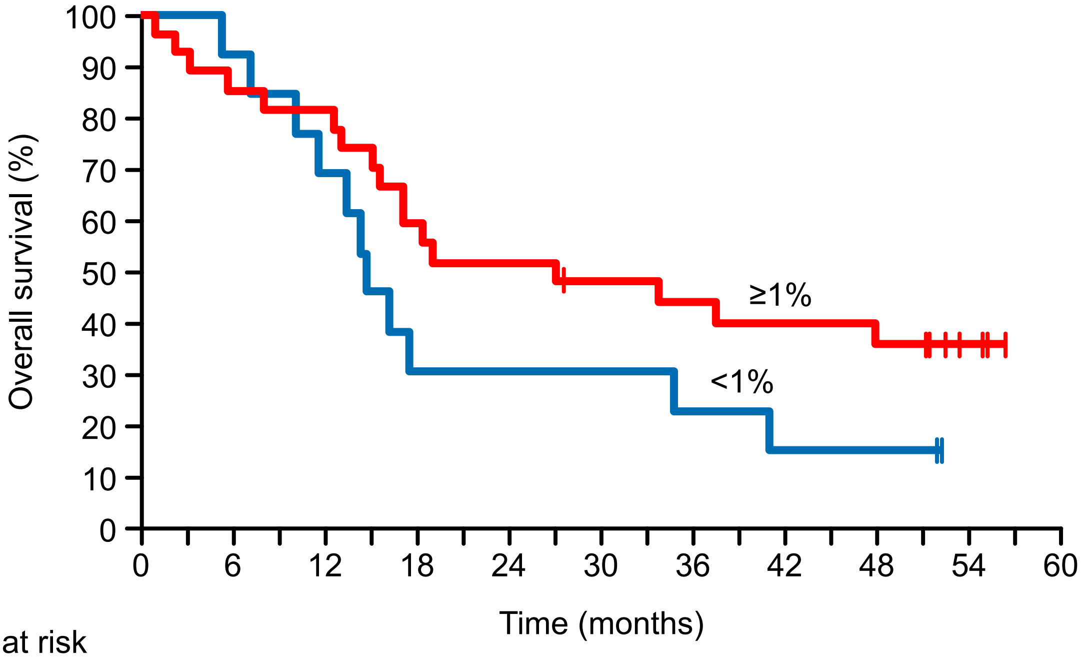

No. at risk
PD-L1

<1%	13	12	9	4	4	4	3	2	2	0
≥1%	27	23	22	16	14	12	11	10	9	4

Figure S4

D

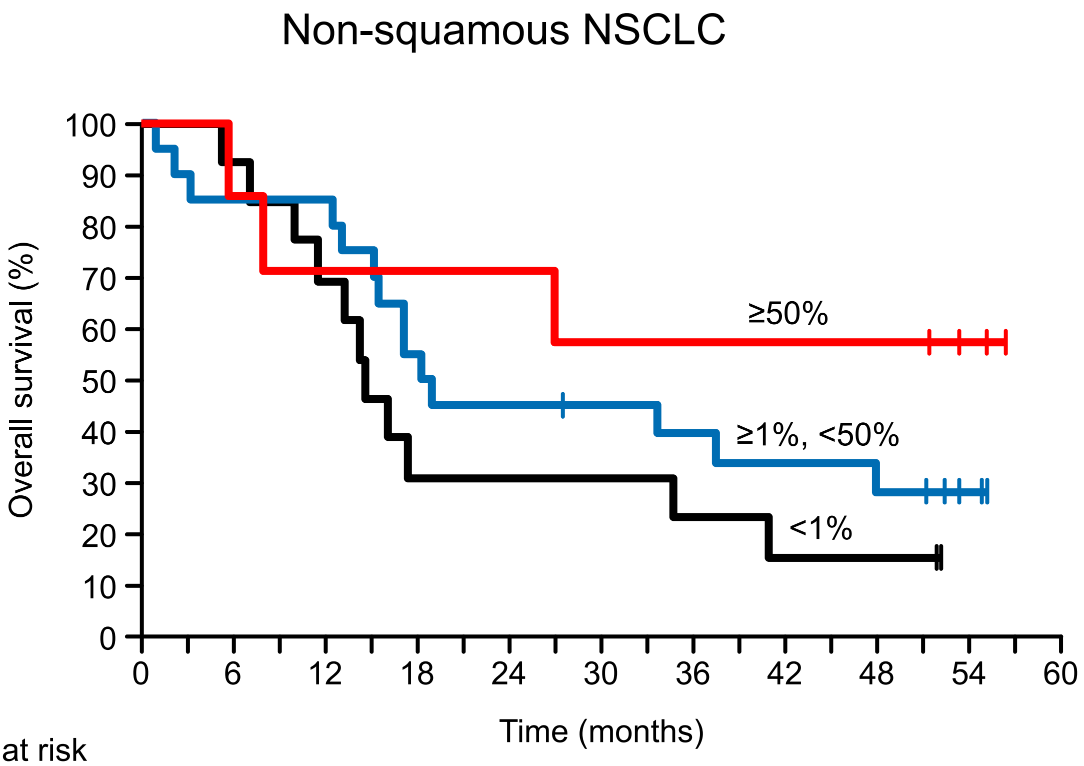

No. at risk
PD-L1

<1%	13	12	9	4	4	4	3	2	2	0
≥1%, <50%	20	17	17	11	9	8	7	6	5	2
≥50%	7	6	5	5	5	4	4	4	4	2

Figure S5. Kaplan–Meier curves of progression-free survival at 3-year follow-up stratified by PD-L1 expression. (A) squamous NSCLC by PD-L1 expression <1% and ≥1%. (B) squamous NSCLC by PD-L1 expression <1%, ≥1% to <50%, and ≥50%. (C) non-squamous NSCLC by PD-L1 expression <1% and ≥1%. (D) non-squamous NSCLC by PD-L1 expression <1%, ≥1% to <50%, and ≥50%.

NSCLC, non-small cell lung cancer; PD-L1, programmed death-ligand 1.

A

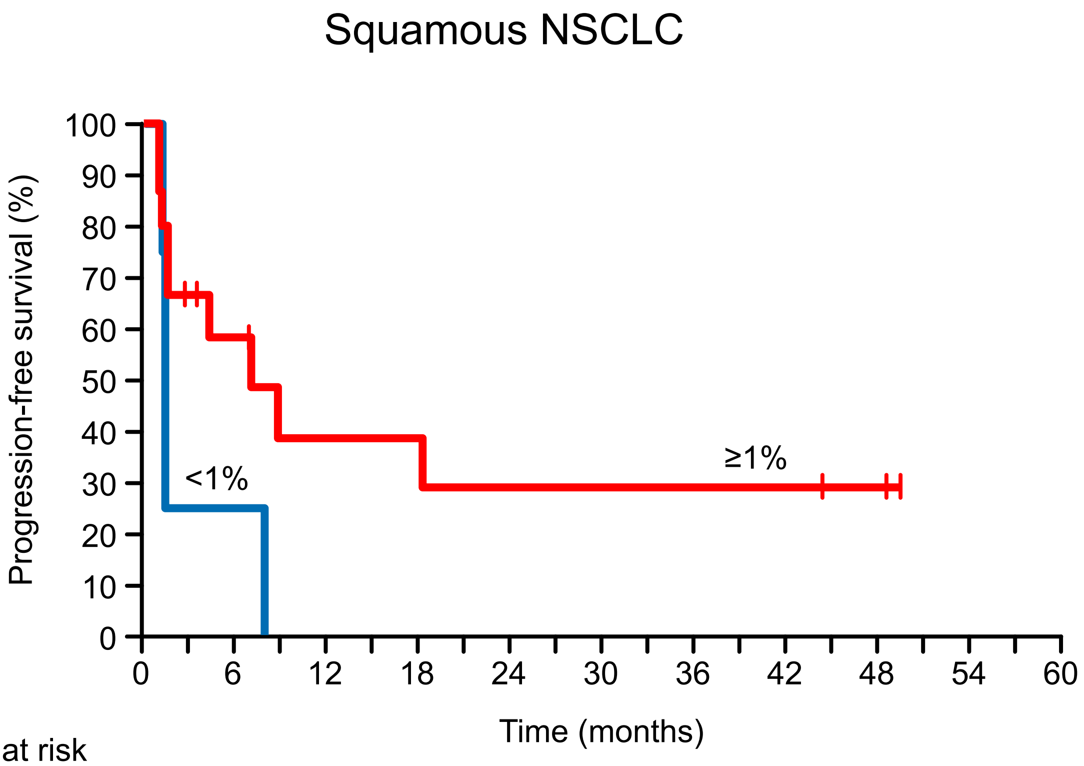

No. at risk PD-L1										
<1%	4	1	0	0	0	0	0	0	0	0
≥1%	15	7	4	4	3	3	3	3	2	0

Figure S5

B

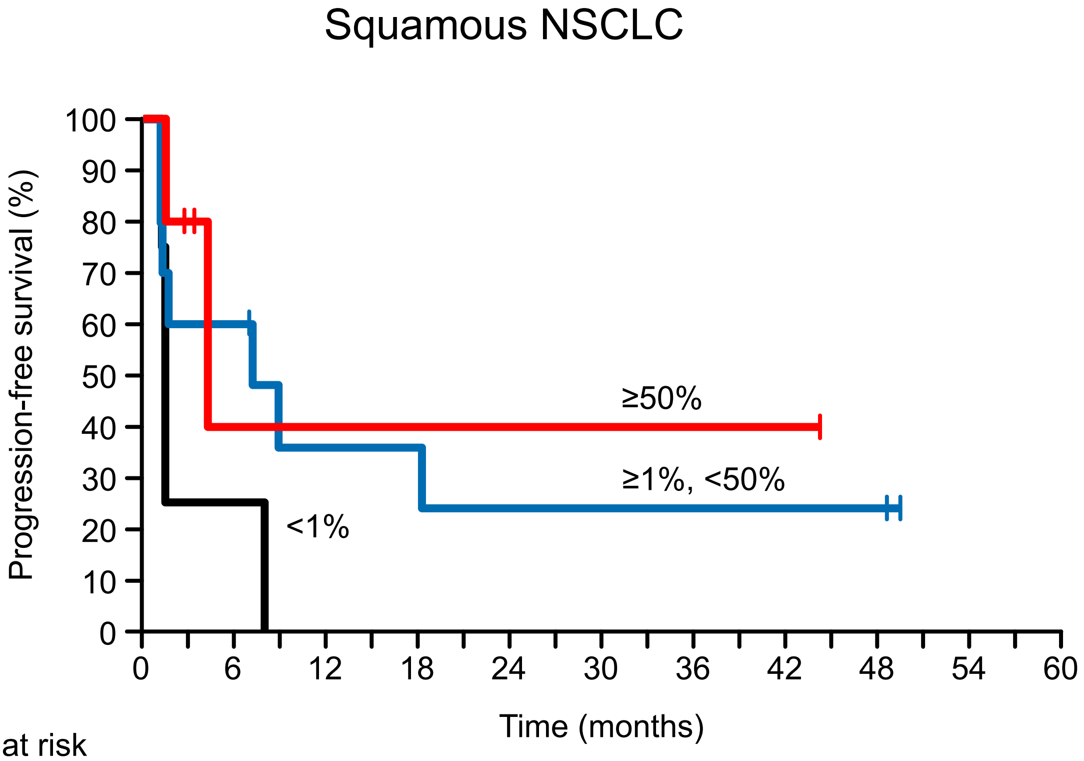

No. at risk
PD-L1

<1%	4	1	0	0	0	0	0	0	0	0
≥1%, <50%	10	6	3	3	2	2	2	2	2	0
≥50%	5	1	1	1	1	1	1	1	0	0

Figure S5

C

Non-squamous NSCLC

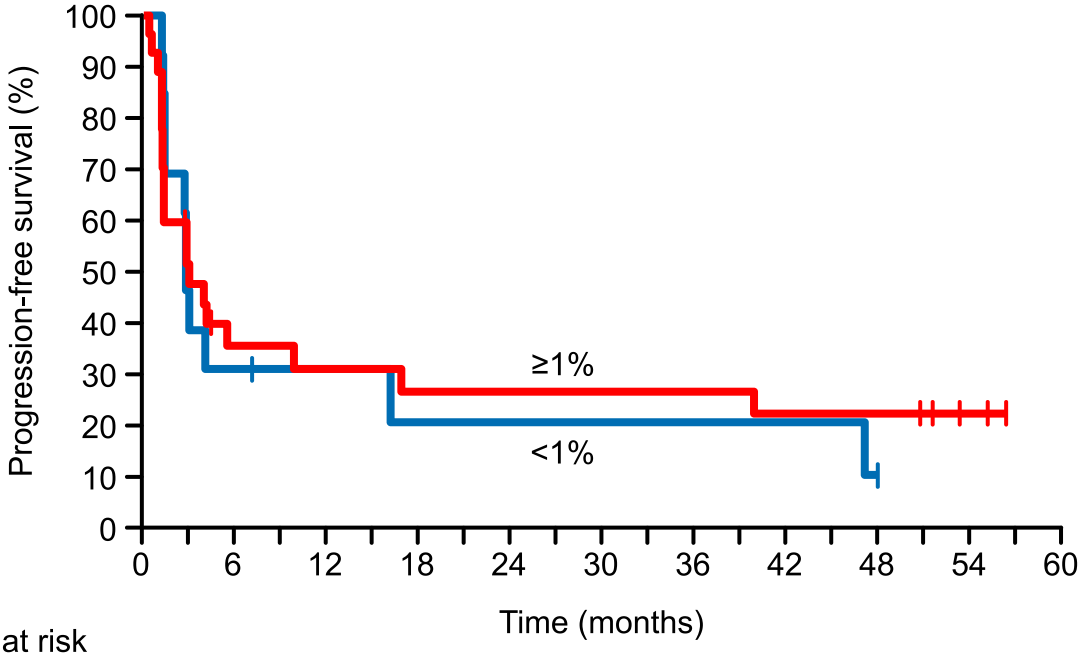

No. at risk

PD-L1

<1%	13	4	3	2	2	2	2	2	0	0
≥1%	27	8	7	6	6	6	6	5	5	2

Figure S5

D

Non-squamous NSCLC

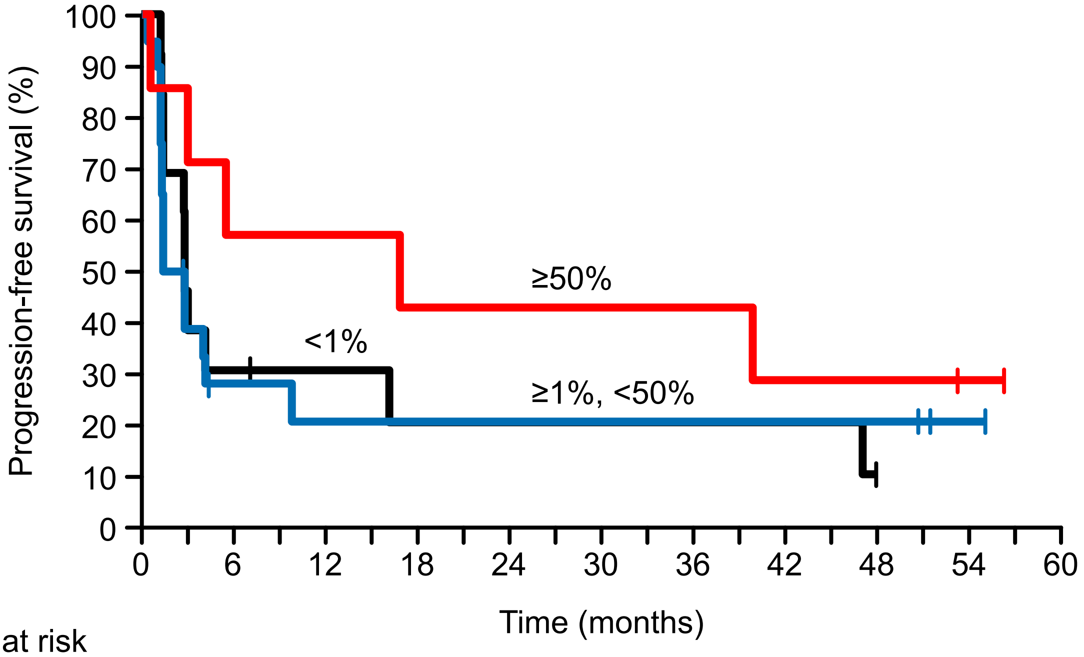

No. at risk
PD-L1

<1%	13	4	3	2	2	2	2	2	0	0
≥1%, <50%	20	4	3	3	3	3	3	3	3	1
≥50%	7	4	4	3	3	3	3	2	2	1
