# Supplementary material for: Three‐year follow‐up results from phase II studies of nivolumab in Japanese patients with previously treated advanced non‐small cell lung cancer: Pooled analysis of ONO‐4538‐05 and ONO‐4538‐06 studies
Source: Cancer Med. 2019 Jul 29;8(11):5183–93. doi: 10.1002/cam4.2411 (PMC6718542; doi:10.1002/cam4.2411)
Supplement: Supplementary file 2 [file CAM4-8-5183-s002.pdf]

**Table S1.** Subsequent chemotherapies received by squamous and non-squamous NSCLC patients after treatment discontinuation.

| Non-SQ NSCLC, N=76 | | | SQ NSCLC, N=35 | | |
|---|---|---|---|---|---|
| | n | % | | n | % |
| Overall | 42 | 55.3 | Overall | 24 | 68.6 |
| Docetaxel | 16 | 21.1 | Docetaxel | 17 | 48.6 |
| Tegafur, gimeracil, oteracil potassium | 6 | 7.9 | Tegafur, gimeracil, oteracil potassium | 2 | 5.7 |
| Pemetrexed sodium hydrate | 3 | 3.9 | Carboplatin + paclitaxel | 2 | 5.7 |
| Erlotinib | 3 | 3.9 | Gemcitabine | 2 | 5.7 |
| Docetaxel + bevacizumab | 2 | 2.6 | TAS-116 | 1 | 2.9 |
| Paclitaxel | 2 | 2.6 | | | |
| Vinorelbine | 2 | 2.6 | | | |
| Tegafur, gimeracil, oteracil potassium + bevacizumab | 2 | 2.6 | | | |
| Paclitaxel + carboplatin | 1 | 1.3 | | | |
| Paclitaxel + bevacizumab | 1 | 1.3 | | | |
| Gefitinib | 1 | 1.3 | | | |
| Crizotinib | 1 | 1.3 | | | |
| Selumetinib (AZD6244) | 1 | 1.3 | | | |
| Investigational drug | 1 | 1.3 | | | |

NSCLC, non-small cell lung cancer; SQ, squamous.

**Table S2.** Summary of efficacy in patients with squamous and non-squamous non-small cell lung cancer with PD-L1 subgrouping analysis.

| | SQ | | | | | Non-SQ | | | | |
|---|---|---|---|---|---|---|---|---|---|---|
| **PD-L1 expression** | **All** | **<1%** | **≥1%** | **1–<50%** | **≥50%** | **All** | **<1%** | **≥1%** | **1–<50%** | **≥50%** |
| **Number of patients** | **N=35** | **N=4** | **N=15** | **N=10** | **N=5** | **N=76** | **N=13** | **N=27** | **N=20** | **N=7** |
| ORR, % | 25.7 (14.2–42.1) | 0.0 (0.0–49.0) | 46.7 (24.8–69.9) | 60.0 (31.3–83.2) | 20.0 (3.6–62.4) | 22.4 (14.5–32.9) | 23.1 (8.2–50.3) | 33.3 (18.6–52.2) | 25.0 (11.2–46.9) | 57.1 (25.0–84.2) |
| DCR, % | 54.3 (38.2–69.5) | 25.0 (4.6–69.9) | 66.7 (41.7–84.8) | 60.0 (31.3–83.2) | 80.0 (37.6–96.4) | 47.4 (36.5–58.4) | 53.8 (29.1–76.8) | 51.9 (34.0–69.3) | 45.0 (25.8–65.8) | 71.4 (35.9–91.8) |
| Median DOR, months (range) | NR (3.0–51.0) | NR (NA–NA) | NR (3.0–48.2) | 12.7 (3.0–48.2) | NR (43.2–43.2) | 41.5 (1.6–53.8) | 45.8 (10.3–46.5) | NR (1.6–53.8) | NR (1.6–53.8) | NR (4.2–52.0) |
| Median PFS, months | 4.2 (1.4–7.1) | 1.4 (1.2–7.9) | 7.1 (1.2–NA) | 7.1 (1.0–NA) | 4.3 (1.6–NA) | 2.8 (1.4–3.4) | 2.8 (1.4–16.1) | 3.0 (1.3–9.8) | 2.1 (1.2–4.1) | 16.9 (0.6–NA) |
| 1-yr PFS, % | 24.5 (10.7–41.3) | 0.0 (NA–NA) | 38.9 (13.2–64.3) | 36.0 (9.0–64.8) | 40.0 (1.1–82.9) | 24.2 (14.9–34.7) | 30.8 (9.5–55.4) | 30.7 (14.3–48.9) | 20.8 (5.9–41.9) | 57.1 (17.2–83.7) |
| 2-yr PFS, % | 16.4 (5.4–32.5) | 0.0 (NA–NA) | 29.2 (7.5–55.6) | 24.0 (3.8–53.7) | 40.0 (1.1–82.9) | 16.1 (8.5–25.9) | 20.5 (3.8–46.3) | 26.3 (11.2–44.4) | 20.8 (5.9–41.9) | 42.9 (9.8–73.4) |
| 3-yr PFS, % | 16.4 (5.4–32.5) | 0.0 (NA–NA) | 29.2 (7.5–55.6) | 24.0 (3.8–53.7) | 40.0 (1.1–82.9) | 14.5 (7.3–24.1) | 20.5 (3.8–46.3) | 26.3 (11.2–44.4) | 20.8 (5.9–41.9) | 42.9 (9.8–73.4) |
| Median OS, months | 16.3 (12.4–25.2) | 14.6 (6.6–18.6) | 12.6 (6.3–25.2) | 17.3 (4.7–27.5) | 12.6 (7.3–NA) | 17.1 (13.3–23.0) | 14.6 (10.0–34.7) | 27.0 (15.1–NA) | 18.6 (13.1–47.9) | NR (5.7–NA) |
| 1-yr OS, % | 71.4 (53.4–83.5) | 75.0 (12.8–96.1) | 60.0 (31.8–79.7) | 60.0 (25.3–82.7) | 60.0 (12.6–88.2) | 68.0 (56.2–77.3) | 69.2 (37.3–87.2) | 81.5 (61.1–91.8) | 85.0 (60.4–94.9) | 71.4 (25.8–92.0) |
| 2-yr OS, % | 37.1 (21.6–52.7) | 0.0 (NA–NA) | 33.3 (12.2–56.4) | 40.0 (12.3–67.0) | 20.0 (0.8–58.2) | 37.4 (26.5–48.1) | 30.8 (9.5–55.4) | 51.9 (31.9–68.5) | 45.0 (23.1–64.7) | 71.4 (25.8–92.0) |
| 3-yr OS, % | 20.0 (8.8–34.4) | 0.0 (NA–NA) | 20.0 (4.9–42.4) | 20.0 (3.1–47.5) | 20.0 (0.8–58.2) | 31.9 (21.7–42.5) | 23.1 (5.6–47.5) | 44.1 (25.2–61.6) | 39.4 (18.6–59.7) | 57.1 (17.2–83.7) |

Data are shown with 95% CIs unless otherwise stated.

CI, confidence interval; DCR, disease control rate; DOR, duration of response; NA, not available; NR, not reached; ORR, overall response rate; OS, overall survival; PD-L1, programmed death-ligand 1; PFS, progression-free survival; SQ, squamous.

**Table S3.** Efficacy summary for pooled squamous and non-squamous NSCLC populations.

| Efficacy parameter | Pooled SQ and non-SQ (N=111) |
|---|---|
| ORR, % | 23.4 (16.5–32.1) |
| DCR, % | 49.5 (40.4–58.7) |
| Median DOR, months (range) | 41.5 (1.6–53.8) |
| Median PFS, months | 2.8 (1.6–4.0) |
| 1-yr PFS, % | 24.5 (16.5–33.4) |
| 2-yr PFS, % | 16.3 (9.6–24.5) |
| 3-yr PFS, % | 15.2 (8.7–23.2) |
| Median OS, months | 17.1 (14.2–20.6) |
| 1-yr OS, % | 69.1 (59.6–76.8) |
| 2-yr OS, % | 37.3 (28.3–46.2) |
| 3-yr OS, % | 28.1 (20.0–36.7) |

Data are shown with 95% CIs unless otherwise stated.

CI, confidence interval; DCR, disease control rate; DOR, duration of response; NSCLC, non-small cell lung cancer; ORR, overall response rate; OS, overall survival; PFS, progression-free survival; SQ, squamous.

**Table S4.** Treatment-related adverse events by MedDRA preferred term after 1 year of nivolumab treatment in squamous and non-squamous non-small cell lung cancer patients.

| | Grade | Non-SQ N=76 | Time to onset (months) | | Grade | SQ N=35 | Time to onset (months) |
|---|---|---|---|---|---|---|---|
| All treatment-related AEs | | 8 (10.5) | | All treatment-related AEs | | 4 (11.4) | |
| Arthralgia | 1 | 3 (3.9) | 14.3, 15.8, 37.7 | Diarrhea | 1 | 2 (5.7) | 13.1, 16.9 |
| Pruritus | 1 | 2 (2.6) | 15.1, 56.1 | Blood creatinine increased | 1 | 2 (5.7) | 14.6, 27.8 |
| Lymphocyte count decreased | 2 | 1 (1.3) | 16.6 | Hypophosphatemia | 3 | 1 (2.9) | 19.2 |
| Diarrhea | 2 | 1 (1.3) | 31.5 | Urinary protein positive | 2 | 1 (2.9) | 15.5 |
| Diarrhea | 1 | 1 (1.3) | 14.1 | Gastritis | 1 | 1 (2.9) | 18.3 |
| Constipation | 1 | 1 (1.3) | 13.7 | Hypersensitivity | 1 | 1 (2.9) | 14.3 |
| Platelet count decreased | 2 | 1 (1.3) | 38.4 | Hyperkalemia | 1 | 1 (2.9) | 15.1 |
| Platelet count decreased | 1 | 1 (1.3) | 26.6 | | | | |
| Enterocolitis | 3 | 1 (1.3) | 32.6 | | | | |
| Amylase increased | 3 | 1 (1.3) | 15.6 | | | | |
| Pharyngitis | 2 | 1 (1.3) | 14.4 | | | | |
| Blood creatine phosphokinase-MB increased | 2 | 1 (1.3) | 15.6 | | | | |
| Blood creatine phosphokinase increased | 2 | 1 (1.3) | 15.1 | | | | |
| Stomatitis | 2 | 1 (1.3) | 44.2 | | | | |
| Hypertension | 2 | 1 (1.3) | 27.8 | | | | |

| | | | |
|---|---|---|---|
| Bacterial pneumonia | 2 | 1 (1.3) | 14.0 |
| Gingivitis | 2 | 1 (1.3) | 22.3 |
| Colitis | 2 | 1 (1.3) | 17.3 |
| Cerebral infarction | 2 | 1 (1.3) | 24.6 |
| Abulia | 2 | 1 (1.3) | 22.4 |
| Cystitis | 2 | 1 (1.3) | 12.4 |
| Acne | 1 | 1 (1.3) | 18.5 |
| Dermatitis acneiform | 1 | 1 (1.3) | 20.6 |
| Nausea | 1 | 1 (1.3) | 15.0 |
| Chest computed tomography abnormal | 1 | 1 (1.3) | 16.5 |
| Cheilitis | 1 | 1 (1.3) | 51.0 |
| Eosinophil count increased | 1 | 1 (1.3) | 19.2 |
| Erythema | 1 | 1 (1.3) | 31.6 |
| Rash maculo-papular | 1 | 1 (1.3) | 36.0 |
| Dry skin | 1 | 1 (1.3) | 42.8 |
| Abdominal pain | 1 | 1 (1.3) | 13.5 |
| Peripheral neuropathy | 1 | 1 (1.3) | 21.4 |

Data are shown as n (%)
SQ, squamous

**Table S5.** Association between treatment-related selected AE incidence and ORR or 1-year OS rate in pooled non-small cell lung cancer patients.

| | N | | ORR | | 1-yr OS rate | | 2-yr OS rate | | 3-yr OS rate | |
|---|---|---|---|---|---|---|---|---|---|---|
| | + | − | + | − | + | − | + | − | + | − |
| All treatment-related selected AE | 55 | 56 | 38.2 (26.5–51.4) | 8.9 (3.9–19.3) | 80.0 (66.8–88.4) | 58.2 (44.1–70.0) | 56.4 (42.3–68.2) | 18.2 (9.4–29.3) | 47.3 (33.7–59.7) | 8.3 (2.8–17.8) |
| Treatment-related selected AE within 2 weeks | 24 | 87 | 37.5 (21.2–57.3) | 19.5 (12.6–29.1) | 87.5 (66.1–95.8) | 64.0 (52.9–73.1) | 58.3 (36.4–75.0) | 31.4 (21.9–41.3) | 45.8 (25.6–64.0) | 23.1 (14.8–32.4) |
| Treatment-related selected AE within 4 weeks | 33 | 78 | 27.3 (15.1–44.2) | 21.8 (14.1–32.2) | 72.7 (54.1–84.8) | 67.6 (55.9–76.8) | 45.5 (28.2–61.2) | 33.8 (23.5–44.3) | 33.3 (18.2–49.3) | 25.8 (16.6–35.9) |
| Treatment-related selected AE within 6 weeks | 39 | 72 | 30.8 (18.6–46.4) | 19.4 (12.0–30.0) | 74.4 (57.6–85.3) | 66.2 (54.0–75.9) | 48.7 (32.5–63.2) | 31.0 (20.7–41.9) | 38.5 (23.5–53.2) | 22.3 (13.4–32.5) |
| Treatment-related selected AE 6-week landmark analysis† | 26 | 50 | 46.2 (28.8–64.5) | 28.0 (17.5–41.7) | 92.3 (72.6–98.0) | 82.0 (68.3–90.2) | 69.2 (47.8–83.3) | 38.0 (24.8–51.1) | 53.8 (33.3–70.6) | 29.6 (17.6–42.5) |
| Treatment-related selected AE Grades 1–2 | 54 | 57 | 38.9 (27.0–52.2) | 8.8 (3.8–18.9) | 81.5 (68.3–89.6) | 57.2 (43.3–68.9) | 57.4 (43.2–69.3) | 17.9 (9.2–28.9) | 48.1 (34.4–60.6) | 8.2 (2.7–17.5) |
| Treatment-related selected AE Grades 3–4 | 6 | 105 | 66.7 (30.0–90.3) | 21.0 (14.3–29.7) | 83.3 (27.3–97.5) | 68.3 (58.4–76.3) | 50.0 (11.1–80.4) | 36.6 (27.4–45.7) | 50.0 (11.1–80.4) | 26.8 (18.7–35.6) |

†Patients maintaining PFS without censoring at 6 weeks were analyzed.

Data are shown as percent with 95% confidence intervals.

AE, adverse event; ORR, overall response rate; OS, overall survival.

**Table S6.** Incidence of treatment-related selected AEs according to baseline characteristics of the pooled squamous and non-squamous non-small cell lung cancer populations.

| | **No treatment-related selected AEs** | **Any treatment-related selected AEs** | **Grades 3−4 treatment-related selected AEs** |
|---|---|---|---|
| | N=56 | N=55 | N=6 |
| Median age (range) | 64 (31–78) | 65 (40–85) | 65 (56–78) |
| Sex, female, % | 30.4 | 23.6 | 33.3 |
| ECOG PS 0, % | 46.4 | 36.4 | 16.7 |
| Brain metastasis, yes, % | 21.4 | 21.8 | 33.3 |
| Prior systemic regimens, 1, % | 73.2 | 89.1 | 100.0 |
| Smoking status, never, % | 25.0 | 14.5 | 0.0 |
| EGFR mutation status, positive, % | 28.6 | 10.9 | 0.0 |
| PD-L1 expression level, % | | | |
| <1% | 26.7 | 31.0 | 20.0 |
| 1–<50% | 56.7 | 44.8 | 40.0 |
| ≥50% | 16.7 | 24.1 | 40.0 |
| Median no. of doses (range) | 6 (1–111) | 9 (1–120) | 11 (1–94) |

AE, adverse event; ECOG PS, Eastern Cooperative Oncology Group performance status; EGFR, epidermal growth factor receptor; PD-L1, programmed death-ligand 1.

**Table S7.** Efficacy and safety of nivolumab according to antinuclear antibody status.

| | **Antinuclear antibody-positive** | **Antinuclear antibody-negative** |
|---|---|---|
| | N=46 | N=65 |
| ORR, % (95% CI) | 23.9 (13.9–37.9) | 23.1 (14.5–34.6) |
| Median PFS, months (95% CI) | 2.8 (1.3–5.5) | 2.8 (1.4–4.0) |
| Median OS, months (95% CI) | 17.1 (12.5–25.2) | 16.3 (13.3–23.8) |
| Treatment-related AE, all grades, % | 80.4 | 81.5 |
| Treatment-related AE, grades 3–4, % | 23.9 | 15.4 |
| Treatment-related serious AE, % | 17.4 | 15.4 |

AE, adverse event; CI, confidence interval; ORR, overall response rate; OS, overall survival; PFS, progression-free survival.

**Table S8.** Treatment-related adverse events by antinuclear antibody status in pooled squamous and non-squamous non-small cell lung cancer populations.

| | **Antinuclear antibody-positive** | | **Antinuclear antibody-negative** | |
|---|---|---|---|---|
| | N=46 | | N=65 | |
| | All grades | Grades 3–4 | All grades | Grades 3–4 |
| Fever | 9 (19.6) | 0 (0.0) | 7 (10.8) | 0 (0.0) |
| Malaise | 7 (15.2) | 0 (0.0) | 9 (13.8) | 0 (0.0) |
| Lymphocyte count decreased | 7 (15.2) | 4 (8.7) | 3 (4.6) | 1 (1.5) |
| Decreased appetite | 6 (13.0) | 0 (0.0) | 10 (15.4) | 1 (1.5) |
| Rash | 6 (13.0) | 0 (0.0) | 10 (15.4) | 0 (0.0) |
| Pruritus | 5 (10.9) | 0 (0.0) | 5 (7.7) | 1 (1.5) |
| Fatigue | 5 (10.9) | 0 (0.0) | 5 (7.7) | 1 (1.5) |
| Vomiting | 5 (10.9) | 0 (0.0) | 0 (0.0) | 0 (0.0) |
| Nausea | 4 (8.7) | 0 (0.0) | 8 (12.3) | 0 (0.0) |
| Diarrhea | 4 (8.7) | 0 (0.0) | 5 (7.7) | 0 (0.0) |
| Arthralgia | 4 (8.7) | 0 (0.0) | 3 (4.6) | 0 (0.0) |
| Erythema | 4 (8.7) | 0 (0.0) | 0 (0.0) | 0 (0.0) |
| Hypothyroidism | 3 (6.5) | 0 (0.0) | 4 (6.2) | 0 (0.0) |
| Platelet count decreased | 3 (6.5) | 0 (0.0) | 2 (3.1) | 0 (0.0) |
| Blood creatine phosphokinase increased | 3 (6.5) | 1 (2.2) | 2 (3.1) | 0 (0.0) |
| Hyponatremia | 3 (6.5) | 1 (2.2) | 1 (1.5) | 1 (1.5) |
| Hypertension | 3 (6.5) | 1 (2.2) | 0 (0.0) | 0 (0.0) |
| Dry skin | 3 (6.5) | 0 (0.0) | 0 (0.0) | 0 (0.0) |
| Peripheral edema | 3 (6.5) | 0 (0.0) | 0 (0.0) | 0 (0.0) |
| Rash maculopapular | 2 (4.3) | 0 (0.0) | 5 (7.7) | 0 (0.0) |
| Dermatitis acneiform | 2 (4.3) | 0 (0.0) | 4 (6.2) | 0 (0.0) |
| Constipation | 1 (2.2) | 0 (0.0) | 6 (9.2) | 0 (0.0) |
| Interstitial lung disease | 1 (2.2) | 1 (2.2) | 4 (6.2) | 1 (1.5) |

Data are shown as n (%)
